# Identifying compartments in ecological networks based on energy channels

**DOI:** 10.1002/ece3.3648

**Published:** 2017-11-28

**Authors:** Lei Zhao, Huayong Zhang, Wang Tian, Xiang Xu

**Affiliations:** ^1^ Research Center for Engineering Ecology and Nonlinear Science North China Electric Power University Beijing China; ^2^ Department of Ecology and Evolutionary Biology and Kansas Biological Survey University of Kansas Lawrence KS USA; ^3^ Department of Life Sciences Imperial College London Ascot UK

**Keywords:** compartmentalization, energy channel, food web, removal effects

## Abstract

It has been confirmed in many food webs that the interactions between species are divided into “compartments,” that is, subgroups of highly interacting taxa with few weak interactions between the subgroups. Many of the current methods for detecting compartments in food webs are borrowed from network theory, which do little to improve our understanding of the mechanisms underpinning them. Therefore, a method based on ecological context is needed. Here, we develop a new method for detecting compartments in food webs based on the reliance of each node on energy derived from basal resources (i.e., producers or decomposers). Additional Monte Carlo simulations were conducted to test the significance of the compartmentalization. Further, we applied a food web dynamics model to test whether the effects of permutation would be retained within a single compartment. The proposed method identified significant compartments in 23 of the 28 empirical food webs that were investigated. We further demonstrated that the effects of node removal were significantly higher within compartments than between compartments. Our methods and results emphasize the importance of energy channels in forming food web structures, which sheds light on the mechanisms of self‐organization within food webs.

## 1 INTRODUCTION

The structure of food webs is believed to affect community stability or species persistence (Dunne, 2006; May, 1973); however, it is still a central challenge to understand how food web structure influences ecosystem functions (Thompson et al., 2012). Many food webs have been showed to consist of compartments, which are subgroups of taxa, with many strong interactions occurring within the subgroups and few weak interactions between the subgroups (Cohen, 1978; Girvan & Newman, 2002; Krause, Frank, Mason, Ulanowicz, & Taylor, 2003; Pimm & Lawton, 1980; Rezende, Albert, Fortuna, & Bascompte, 2009; Yodzis, 1982), although the mechanisms of the compartmentalization remain underexplored. The idea of compartmentalization can be traced back to Herbert Simon's (1962) parable of watchmakers, where he showed that partitioning assembly is more efficient and argued that complex systems in nature could benefit from their hierarchical structure. For example, food webs are comprised of many energy flows. The coupling of these flows has been shown to promote food web stability (Rooney, McCann, Gellner, & Moore, 2006; Teng & McCann, 2004). In subsequent years, ecologists have used a wide variety of methods and definitions to search for compartments in food webs (Allen & Starr, 1982; Allesina, Bodini, & Bondavalli, 2005; Borrett, Fath, & Patten, 2007; May, 1972; Pimm, 1979; Pollierer, Langel, Scheu, & Maraun, 2009; Raffaelli & Hall, 1992), many of which are borrowed from network theory.

In network theory, several methods have been developed in recent decades to detect community (i.e., compartment) structure in complex networks (Guimerà, Sales‐Pardo, & Amaral, 2007; Newman, 2004a; Thébault, 2013). The basic methodological idea is to find a division of the network that maximizes modularity, which is a numerical index of how good a particular division is (Newman, 2004a). Modularity measures the fraction of within‐community links minus the expected value of the same quantity in a network with the same community divisions but random connections between the nodes (Newman & Girvan, 2004). Using the methods, compartments have been detected in different kinds of networks, such as social networks (Newman & Girvan, 2004), the Internet (Simonsen, Maslov, Sneppen, & Eriksen, 2003), and biochemical networks (Ravasz, Somera, Mongru, Oltvai, & Barabási, 2002). Ecologists have tried to apply these methods to ecological networks (Krause et al., 2003; Leger, Daudin, & Vacher, 2015; Stouffer & Bascompte, 2011); however, food webs have at least two special characteristics: (1) The distribution of the link weights is largely uneven (O'Gorman, Jacob, Jonsson, & Emmerson, 2010); and (2) food web links have explicit directions from low to high trophic levels. These characteristics provide a cautionary note when interpreting results using methods designed for undirected and unweighted networks (Zhao et al., 2016). Moreover, the methods borrowed from network theory do little to improve our understanding of the underlying mechanisms of compartmentalization in food webs. Therefore, a method based on ecological context is urgently needed.

An energy channel is a collection of energy or material fluxes that start with a basal resource and end with a top predator (Moore & de Ruiter, 2012), which plays an important role in maintaining both food web structure and functioning. Different energy channels within the same food web may exhibit different traits, for example turnover rate (Rooney et al., 2006). For example, in marine food webs, phytoplankton energy channels were shown to have consistently higher turnover rates than detrital energy channels (Rooney, McCann, & Moore, 2008). The coupling of fast (e.g., phytoplankton) and slow (e.g., detrital) channels is believed to contribute to food web stability (Rooney & McCann, 2012; Rooney et al., 2006, 2008). Based on taxonomical aggregation, two compartments were defined: the detritus‐based “brown” subweb and the producer‐based “green” subweb (Butler, Gotelli, & Ellison, 2008; Zou, Thébault, Lacroix, & Barot, 2016). However, this separation is oversimplistic, and the compartmentalization based on energy channels is not fully explored. Here, we propose a new method for detecting compartments, where we consider one or several energy channels as a compartment. Our first task in this study was to test whether empirical food webs could be significantly compartmentalized using our method; that is, whether the modularity of the compartmentalization was significantly higher than what was likely to occur by chance.

Theoretically, compartmentalization is believed to greatly increase the stability and persistence of food webs (Krause et al., 2003; Pimm, 1979; Stouffer & Bascompte, 2011). One possible mechanism is that the impacts of a disturbance can be buffered by being retained within a single compartment and rarely spread to other compartments (Krause et al., 2003), although this hypothesis has not been tested well. In the study by Krause et al. (2003), an assumption was made to mimic the effects of species loss: After a taxon was removed, all interactions with this taxon were removed, and the predators on this taxon had their interaction strengths associated with this taxon redistributed proportionally to their interactions with other prey. This assumption is simple (as admitted by Krause et al.). It cannot track the biomass change after taxon removal, as well as the change in interaction strength associated with the biomass change. Moreover, they compared the removal effects of only two taxa, which may underrepresent the overall pattern. Our second task was to test the hypothesis that compartmentalization could increase food web stability using a dynamical approach. Finally, we compared our algorithm based on energy channels with some other commonly used compartment detection algorithms.

## 2 MATERIALS AND METHODS

### 2.1 Quantitative food webs

We analyzed 28 of the 50 aquatic food webs from a recently published database (see Table 1; Salas & Borrett, 2011; Borrett, 2013), which is archived in the “*enaR”* package (Borrett & Lau, 2014) in R. Twenty food webs were excluded because they contained less than twenty taxa and thus were deemed too small. The other two food webs were excluded because they did not include microbial processes. The data for each food web include a list of taxa, the carbon biomass of each taxon (g C m^−2^), the carbon per unit time of import, export, and respiration of each taxon (g C m^−2^ day^−1^), and the carbon flux between a pair of taxa (g C m^−2^ day^−1^). Our focal food webs exhibit a wide range of taxon richness (*S = *23–124). Nodes represent species, trophic guilds, functional groups, or nonliving components of the system in which matter is stored. The ecosystems were considered to be in steady state, as in previous studies (Borrett, 2013; Borrett & Salas, 2010; Ulanowicz, 2004), which means that the flows of energy entering and leaving a given node are equal. Due to the errors and approximations associated with some measures, however, some food webs were initially unbalanced, that is, energy entering a taxon did not necessarily balance the output exactly. These food webs were balanced using the AVG2 algorithm using established procedures in Matlab 7.12.0 (Allesina & Bondavalli, 2003).

**Table 1:** Compartment analysis of 28 empirical food webs. For each food web, *S* is the number of taxa; *S*_C_ is the number of detected compartments; *Q* is the empirical modularity; *p* is the probability that the modularity of a simulated food web in Monte Carlo simulations is higher than *Q*; and the last two columns indicate the mean and standard error (*SEM*) of modularity in Monte Carlo simulations. Significant *Q* values are marked in bold, where the significance level was α = 0.05/28 = 0.0018 after Bonferroni correction

Index	Name	*S*	*S*_c_	*Q*	*p*	Monte Carlo	
						Mean	*SEM*
1	Chesapeake Bay	36	3	**0.39**	**<.001**	−0.0052	0.0045
2	Cypress (dry)	68	3	0.13	.006	0.0004	0.0005
3	Cypress (wet)	68	3	0.15	.009	−0.0002	0.0005
4	Florida Bay (dry)	124	4	**0.10**	**<.001**	0.0081	0.0034
5	Florida Bay (wet)	124	4	**0.10**	**<.001**	0.0008	0.0035
6	Georges Bank	31	2	**0.10**	**<.001**	−0.0020	0.0033
7	Graminoids (dry)	66	3	**0.05**	**<.001**	0.0019	0.0023
8	Graminoids (wet)	66	3	**0.03**	**<.001**	0.0008	0.0017
9	Gulf of Maine	31	2	**0.10**	**.001**	−0.0006	0.0033
10	Lake Oneida (pre‐ZM)	74	2	**0.08**	**<.001**	−0.0008	0.0004
11	Lake Oneida (post‐ZM)	76	3	0.08	.018	−0.0007	0.0020
12	Mangroves (dry)	94	3	**0.02**	**<.001**	−0.0009	0.0005
13	Mangroves (wet)	94	3	**0.03**	**<.001**	0.0001	0.0005
14	Middle Atlantic Bight	32	2	**0.10**	**.001**	−0.0013	0.0029
15	Narragansett Bay	32	2	0.10	.019	−0.0026	0.0025
16	Neuse Estuary (early summer 1997)	24	3	**0.27**	**<.001**	0.0100	0.0059
17	Neuse Estuary (late summer 1997)	27	3	**0.29**	**<.001**	−0.0009	0.0069
18	Neuse Estuary (early summer 1998)	24	3	**0.33**	**<.001**	−0.0063	0.0060
19	Neuse Estuary (late summer 1998)	25	3	**0.24**	**<.001**	−0.0057	0.0063
20	Northern Benguela Upwelling	23	2	0.08	.004	0.0054	0.0044
21	Southern New England Bight	33	2	**0.11**	**<.001**	−0.0028	0.0034
22	St. Marks Seagrass, site 1 (January)	43	4	**0.20**	**<.001**	0.0018	0.0021
23	St. Marks Seagrass, site 1 (February)	45	4	**0.16**	**<.001**	−0.0003	0.0024
24	St. Marks Seagrass, site 2 (January)	40	3	**0.19**	**<.001**	−0.0004	0.0020
25	St. Marks Seagrass, site 2 (February)	46	3	**0.17**	**<.001**	−0.0015	0.0022
26	St. Marks Seagrass, site 3 (January)	32	4	**0.21**	**.001**	−0.0011	0.0024
27	St. Marks Seagrass, site 4 (February)	46	3	**0.20**	**<.001**	−0.0028	0.0033
28	Sylt‐Romo Bight	59	5	**0.20**	**<.001**	0.0026	0.0033

### 2.2 Measure of modularity

The classic definition of modularity proposed by Newman (2004a,b) was extended by Arenas, Duch, Fernández, and Gómez (2007) to the scenario of weighted directed networks as follows:

(1) $$Q=\frac{1}{2W}\sum_{i}\sum_{j}\left(w_{\mathrm{ij}}-\frac{w_{i}^{\mathrm{out}}w_{j}^{\mathrm{in}}}{2W}\right)\delta \left(c_{i},c_{j}\right)$$

where *w*_*ij*_ is the strength of the link (i.e., the carbon flux) from *i* to *j*, and *w*_*j*_^in^ and *w*_*i*_^out^ are the input and output strengths of nodes *i* and *j*, respectively:

(2) $$w_{i}^{\mathrm{out}}=\sum_{j}w_{\mathrm{ij}}$$

(3) $$w_{j}^{\mathrm{in}}=\sum_{i}w_{\mathrm{ij}}$$

The total strength of the link is:

(4) $$2W=\sum_{i}w_{i}^{\mathrm{out}}=\sum_{j}w_{j}^{\mathrm{in}}=\sum_{i}\sum_{j}w_{\mathrm{ij}}$$

The Kronecker delta function δ (*c*_*i*_, *c*_*j*_) takes the value of 1 if *c*_*i*_ = *c*_*j*_ and otherwise 0, where *c*_*i*_ is the community to which node *i* is assigned. Nonzero values of *Q* indicate deviations from randomness, and a higher value of *Q* indicates the food web is more compartmentalized.

### 2.3 Compartment identification

**Figure 1:**
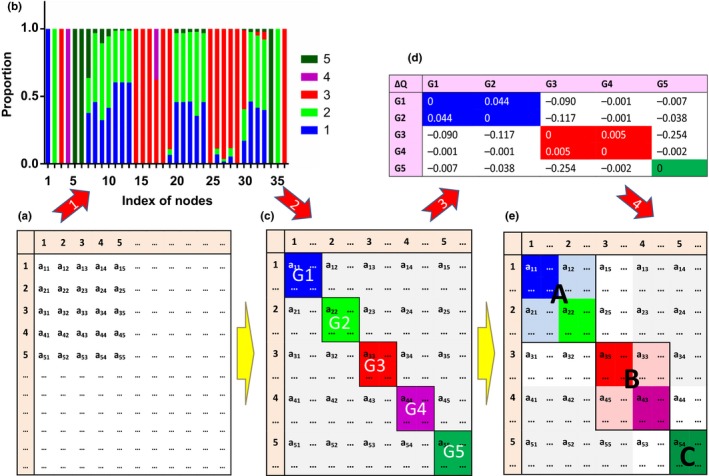
Illustration of the method used to detect compartments in a food web. Panels (a), (c), and (e) show how a simplified feeding matrix changes at different stages of the compartmentalization procedure; the rows correspond to resources, while the columns equate to consumers. The first step of the procedure is to calculate the proportion of energy gained from different basal resources by each node in the food web, as shown in panel (b), where the colors correspond to the five different basal resources. All nodes with the same dominant energy component are placed in the same subgroup, which is G1–G5 in this example, as shown in panel (c). The next step is to calculate Δ*Q* values for possible merging of subgroups, as shown in panel (d). Finally, two subgroups are merged into one compartment if Δ*Q* > 0, as shown in panel (e). Three compartments (A, B, and C) were detected in this simplified example from Chesapeake Bay

Four steps were applied in our method to detect the compartments of a food web. First, a food web with *S* taxa can be represented by an *S*‐by‐*S* flow matrix ***F ***= [*F*_*ij*_]; the value of the element *F*_*ij*_ means the amount of carbon passing from taxon *i* to taxon *j* per unit area and time. Based on the flow matrix (Figure 1a), we calculated the reliance of each node on energy derived from producers or decomposers, that is, the proportion of carbon derived from basal nodes, %*BR*_*C*_ (Figure 1b), using a method proposed by Rooney et al. (2008):

(5) $$\%{\mathrm{BR}}_{C}=\sum_{1}^{n}P_{C}\times \%{\mathrm{BR}}_{R}$$

where *n* is the number of resources consumed by the consumer, *P*_C_ is the proportion of the consumer diet accounted for by a resource, and %*BR*_*R*_ is the proportion of carbon derived from the basal resource in the resource being consumed.

Second, the nodes with the same dominant energy origins were put into the same subgroup. For example, in the Chesapeake Bay ecosystem, five subgroups were formed (Figure 1c). Here, each subgroup corresponds to one of the five basal nodes, and the nodes in a given subgroup obtained most of their energy from the same basal node.

Third, we tested whether the subgroups could be optimized by merging them into one compartment. Here, we calculated the difference in modularity Δ*Q* = *Q*_after_–*Q*_before_, where *Q*_before_ and *Q*_after_ equate to the modularity before and after merging, respectively. By trying different combinations of subgroup pairs, we created a matrix, with the element Δ*Q*_*ij*_ indicating the difference in modularity after merging subgroup *i* and subgroup *j* minus modularity before merging them (Figure 1d).

Fourth, if Δ*Q* > 0, the merging was accepted as it could increase *Q* and otherwise, the merging was rejected (Figure 1e). Repeat steps 3 and 4 until all values of Δ*Q *≤ 0. With this four‐step method, we were able to detect the number of compartments and nodes within them for all the 28 food webs that we investigated.

### 2.4 Monte Carlo simulations

To test whether the concentration of interactions within identified compartments was greater than what was likely to occur by chance, we determined the statistical significance of the modularity using Monte Carlo simulations. For each simulation, we randomly reassigned interactions, constraining the sum of each column in the carbon‐flow matrix to be equal to that of the original food web (where rows represented prey and columns represented predators), as in Krause et al. (2003). The detailed approach is as follows: (1) for column *i* (i.e., for predator *i*), we first calculated the sum of the interaction weight *W*_*i*_; (2) to determine the prey of predator *i* in the new food web, we chose *n*_*i*_ prey randomly from a pool of *S*−1 species where *n*_*i*_ is the number of prey for predator *i* in the original food web, and *S*−1 is the number of species excluding predator *i*; (3) we generated *n*_*i*_ random numbers, *x*_1_, *x*_2_, …, *x*_*ni*_, each of which varied from 0 to 1; and (4) the weight of interactions between the *j*th prey and predator *i* should be $W_{i}x_{j}/\sum_{1}^{n_{i}}x_{t}$. This ensured that the simulated food webs had the same number of predators, the same number and weight of interactions associated with a predator, and the same number and weight of realized interactions as the original food web. We then calculated the modularity of the simulated food web on the compartments obtained from the method we proposed. This process was repeated 1,000 times to obtain a sampling distribution of modularity against which we compared the empirical modularity.

### 2.5 Food web dynamics

To test the hypothesis that compartmentalization would retain the impacts of a disturbance within a single compartment and with less spread to other compartments, we simulated the effects of randomly removing one node, by employing a food web dynamics model (Zhang, Zhao, Tian, & Huang, 2016; Zhao et al., 2016). This model was constructed based on energy budgets indexing the carbon fluxes entering and leaving each taxon. The taxa in each of the studied ecosystems can be divided into four categories: producers, consumers, decomposers, and detritus.

The change in biomass of producers can be described as:

(6) $$\frac{dB_{i}}{dt}=r_{i}B_{i}\left(1-\sum_{j=\mathrm{pro}}B_{j}/K\right)-\sum_{j=\mathrm{herbi}}\Phi_{\mathrm{ij}}B_{j}-d_{i}B_{i}$$

Here, “pro*”* are producer taxa, “herbi*”* are herbivorous taxa, *r* is the maximum specific or intrinsic growth rate, *K* is the carrying capacity, *d* is the specific death rate, and Φ_*ij*_ is the functional response when taxon *j* consumes taxon *i*, which was set to follow a nonlinear form as follows (Hudson & Reuman, 2013):

(7) $$\Phi_{\mathrm{ij}}=\frac{y_{j}\omega_{\mathrm{ij}}B_{i}^{h}}{H_{j}^{h}+q_{j}B_{j}H_{j}^{h}+\sum_{k=\mathrm{res}}\omega_{\mathrm{kj}}B_{k}^{h}}$$

Here, *y*_*j*_ is the maximum consumption rate of taxon *j*, ω_*ij*_ is the preference of taxon *j* for taxon *i*,* H*_*j*_ is the half‐saturation density, *q*_*j*_ is the predator interference coefficient, and *h* is the hill exponent that regulates the shape of the curve from Holling type II (*h *=* *1) to Holling type III (*h *=* *2).

The change in biomass of consumers (including herbivores and predators) can be depicted as:

(8) $$\frac{dB_{i}}{dt}=\sum_{j=\mathrm{res}}a_{i}\Phi_{\mathrm{ji}}B_{i}-\sum_{j=\mathrm{pred}}\Phi_{\mathrm{ij}}B_{j}-x_{i}B_{i}$$

where, “res” are resource taxon, “pred” are predatory taxon, *a* is the assimilation efficiency, and *x* is the respiration rate.

The change in biomass of decomposers can be depicted as:

(9) $$\frac{dB_{i}}{dt}=\sum_{j=\det}a_{i}\Phi_{\mathrm{ji}}B_{i}-\sum_{j=\mathrm{pred}}\Phi_{\mathrm{ij}}B_{j}-x_{i}B_{i}$$

where “det” are detrital taxa.

In some food webs, detritus has been divided into separate taxa. The change in biomass of each detrital taxa can be described as:

(10) $$\frac{dB_{i}}{dt}=\sum_{j=\mathrm{pro}}p_{\mathrm{ji}}d_{j}B_{j}+\sum_{j=\mathrm{con}}\left(p_{\mathrm{ji}}e_{j}B_{j}\sum_{k=\mathrm{res}}\Phi_{\mathrm{kj}}\right)+\sum_{j=\det}c_{\mathrm{ji}}B_{j}-\sum_{j=\mathrm{dec}}\Phi_{\mathrm{ij}}B_{j}-\sum_{j=\det}c_{\mathrm{ij}}B_{i}$$

Here, “con” are consumer taxa, “dec” are decomposer taxa, *p*_*ji*_ is the proportion of converted detritus *i* to the total amount of detritus converted from taxon *j*,* e = *(1–*a*) is the egestion rate, and *c*_*ji*_ is the conversion coefficient from detrital taxon *j* to detrital taxon *i*. Here, we consider that the amount of feces, that is, the unassimilated fraction of prey killed, is proportional to the amount of predation (Moore & de Ruiter, 2012; Moore, De Ruiter, & Hunt, 1993; de Ruiter, Neutel, & Moore, 1995).

The setting and calculation of the parameters from carbon flux data can be found in Appendix S1 and Table S1. We used the Adaptive Runge–Kutta method to perform 1,000 numerical simulations for each food web. In each simulation, the empirical biomass data were employed to give the initial biomass values. First, 1,000 days were simulated to allow transient dynamics caused by initial effects to settle down (Hudson & Reuman, 2013). The average biomass density for each node during the next 1,000 simulated days was recorded as *B*_*i*_^*+*^. We then randomly removed a node and simulated another 1,000 days, recording the average biomass density for each node during this period as *B*_*i*_^*−*^.

### 2.6 Removal effects

The removal effect *(RE) value of the* loss of node *k* on node *i* was measured according to Brose, Berlow, and Martinez (2005) as:

(11) $${\mathrm{RE}}_{i}=\log\left[\left(B_{i}^{+}+1\right)/\left(B_{i}^{-}+1\right)\right]/\left(B_{k}+1\right)$$

where *B*_*k*_ is the biomass density of the node to be removed before its removal, while *B*_*i*_^*+*^ and *B*_*i*_^*−*^ are the biomass densities of *i* with and without *k*, respectively. Adding one to the biomass densities prevents very low *B*_*k*_ from generating artificially large values of RE (Brose et al., 2005). The within‐compartment RE is the average RE value for all the nodes within the same compartment as *k*, and the between‐compartment RE is the average RE value for all the nodes in different compartments to *k*.

### 2.7 Comparison with other algorithms

To test the performance of our algorithm based on energy channels, we compared the modularity values that were generated with two other commonly used algorithms. The Girvan–Newman algorithm (Newman & Girvan, 2004) first estimates the edge betweenness score as the number of shortest paths through an edge. This algorithm then gradually removes the edge with the highest edge betweenness score to produce a dendrogram of removals. This dendrogram is then cut at the point which gives the highest value of modularity. The second algorithm finds densely connected subgraphs by performing random walks (Pons & Latapy, 2005), where each walk tends to stay inside communities instead of jumping to other communities.

### 2.8 Statistical analyses

To compare the two categories of RE values (within‐compartment, and between‐compartment), we employed paired *t* test. To compare the performance of the three compartmentalization algorithms, we employed a linear mixed effects model (LME) with a maximum‐likelihood estimator (function “*lme”* with “*method* = *ML”* within the “*nlme”* package in R). Food web identity was included in the model as a random factor to correct for differences between study systems. Post hoc comparisons were applied using the Tukey HSD test at α = 0.05 level of significance (function “*glht”* within the “*multcomp”* package in R).

## 3 RESULTS

**Figure 2:**
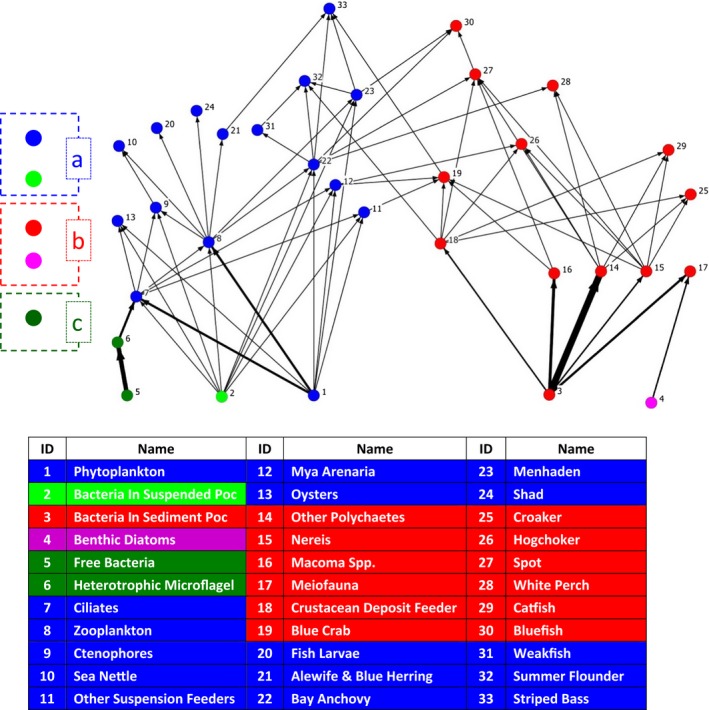
Graphical display of the compartments in the Chesapeake Bay ecosystem. Each of the numbered node indicates a taxon, with names corresponding to the numbers listed in the table. Links between the 36 taxa are weighted by carbon flux, while the various colors indicate different compartments

First, we showed a detailed example of one food web: the Chesapeake Bay. This ecosystem contained 31 consumer and decomposer taxa and five basal taxa: phytoplankton, benthic diatoms, dissolved organic nutrient, suspended particulate nutrient, and sediment particulate nutrient (Figure 2). Among all the 31 consumer and decomposer taxa, the energy of 13 taxa was mainly supplied by sediment particulate nutrient, while suspended particulate nutrient was the major supplier for another 12 taxa (Figure 1b), phytoplankton was the major supplier for four taxa, dissolved organic nutrient was the major supplier for two taxa, and benthic diatoms were not a major supplier for any taxa. Three compartments were detected, one of which was mainly sustained by suspended particulate nutrient and phytoplankton; another compartment was mainly sustained by sediment particulate nutrient but also contained benthic diatoms, while the final compartment contained just three taxa: free bacteria, heterotrophic microflagellates, and dissolved organic nutrient.

The compartments of all 28 food webs (Appendix S2) showed three types of compartmentalization. In the first type, pelagic taxa (supported by phytoplankton) and benthic taxa (supported by sediment bacteria or benthic algae) were separated. Sometimes, the taxa supported by sediment bacteria were also separated from the taxa supported by benthic algae. Fifteen ecosystems showed this type of compartmentalization, including Chesapeake Bay, Georges Bank, Gulf of Maine, Lake Oneida (pre‐ZM), Lake Oneida (post‐ZM), Mangroves (dry), Mangroves (wet), Middle Atlantic Bight, Narragansett Bay, Neuse Estuary (early summer 1997), Neuse Estuary (late summer 1997), Neuse Estuary (early summer 1998), Neuse Estuary (late summer 1998), Northern Benguela Upwelling, and Southern New England Bight. The second type consisted of three compartments, containing taxa supported by macrophytes, phytoplankton, or sediment bacteria. Eleven ecosystems showed this type of compartmentalization, including Florida Bay (dry), Florida Bay (wet), Graminoids (dry), Graminoids (wet), St. Marks Seagrass, site 1 (January), St. Marks Seagrass, site 1 (February), St. Marks Seagrass, site 2 (January), St. Marks Seagrass, site 2 (February), St. Marks Seagrass, site 3 (January), St. Marks Seagrass, site 4 (February), and Sylt‐Romo Bight. The third type of compartmentalization separated aquatic from terrestrial communities, corresponding to just two ecosystems: Cypress (dry) and Cypress (wet).

We detected between two and five compartments in each of the 28 food webs. Over 80% of them yielded modularity that was significantly greater than would be expected by chance alone (Table 1). The modularity *Q* varied from 0.02 to 0.39, while the modularity values in Monte Carlo simulations varied from −0.006 to 0.010.

**Figure 3:**
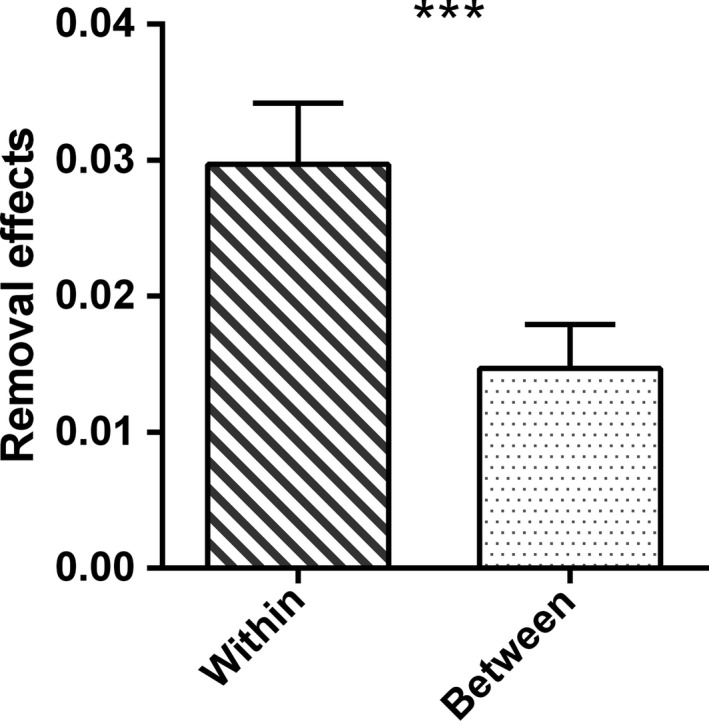
Removal effects (*RE*: mean ± *SEM*) of the 28 food webs. Two categories of *RE* are displayed: within‐compartment *RE* and between‐compartment *RE*. Significant difference between the categories is indicated by stars (*** *p *<* *.001), detected using paired *t* test

In the node deletion experiment (Figure 3), the within‐compartment RE exhibited higher value (0.030 ± 0.004; mean ± *SEM*) than between‐compartment RE (0.015 ± 0.003), and the difference is significant (paired *t* test: *t*_27_ = 3.727, *p *<* *.001).

**Figure 4:**
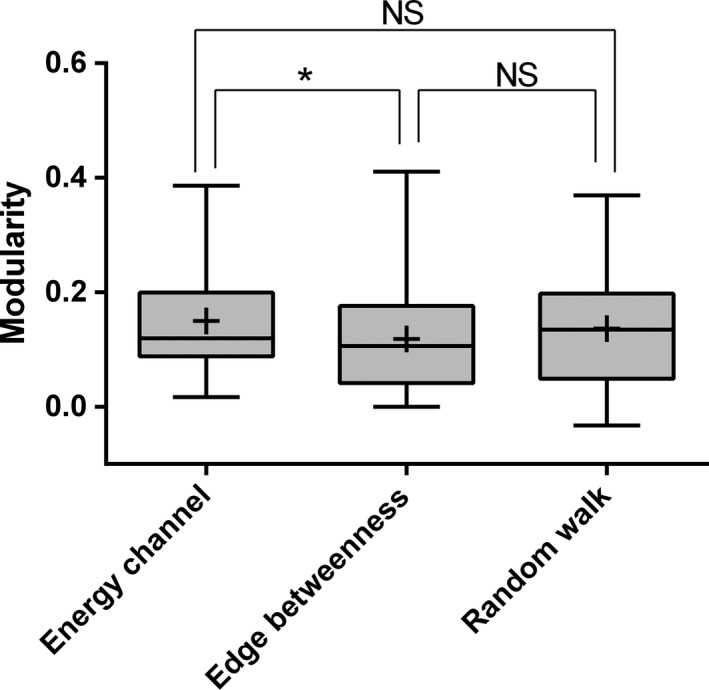
Modularity of the 28 food webs after compartmentalization according to three different algorithms: energy channel, edge betweenness, and random walk. Significant differences between pairs of categories are indicated by stars on lines connecting the pairs (*p <* *.05; NS, not significant), detected using LME and Tukey post hoc tests

Our algorithm based on energy channels generated the highest modularity (0.150 ± 0.017; mean ± *SEM*) across the 28 food webs, compared with algorithms of edge betweenness (0.119 ± 0.020) and random walk (0.137 ± 0.019). Different algorithm generated significantly different modularity (LME: *F*_2,54_ = 3.584, *p *=* *.035; Figure 4). Here, our algorithm generated significantly higher modularity than the algorithm of edge betweenness (Tukey test: *z *=* *2.667, *p *=* *.023), but not the algorithm of random walk (Tukey test: *z *=* *1.128, *p *=* *.778).

## 4 DISCUSSION

Most food webs in our study were found to be strongly compartmentalized, in agreement with previous research (Guimera et al., 2010; Krause et al., 2003; Rezende et al., 2009). The compartmentalization of food webs has been claimed to arise from subhabitats within the environment (Krause et al., 2003) or because of phylogenetic patterns within the community (Cattin, Bersier, Banašek‐Richter, Baltensperger, & Gabriel, 2004; Rezende et al., 2009). Here, we demonstrated that energy channels could be another origin of compartmentalization. Being compartmentalized is to a community's advantage as compartments act to buffer the effects of perturbation.

Energy channels derived from producers and detritus (namely “green” and “brown” food webs) have been investigated in recent studies (Butler et al., 2008; Rooney & McCann, 2012; Rooney et al., 2006, 2008). Most of these studies focused on the coupling of the two channels, which was believed to increase the stability of ecosystems. In these studies, phytoplankton and detritus were chosen as the origin of energy, and each food web was divided into two subwebs based on their energy origin; however, this might be an oversimplification. Based on our results, different types of detritus may sustain very different compartments. For example, suspended detritus and sediment detritus sustained different compartments in most of our 28 ecosystems. On the other hand, phytoplankton may sustain the same compartment with some types of detritus (e.g., suspended detritus or dissolved organic carbon). Using a more elaborate partition of energy channels may provide new insights into studying the coupling of channels.

Across all 28 food webs in our study, the number of compartments varied from two to five. This is highly consistent with prior studies, where these numbers varied from one to six across 17 food webs (three of them overlap with ours; Krause et al., 2003) or from two to three across nine food webs (one of them overlap with ours; Gauzens, Thébault, Lacroix, & Legendre, 2015). However, such small number did not provide support for the original argument of complex system assembly, suggested by Simon (1962). We attribute the small number of compartments to three reasons. First, the level of taxonomical aggregation should have an influence, especially for basal taxa. For example, suspended detritus and sediment detritus were separated in most food webs and in food webs where they were not separated, and detritus was considered as a single node; that is, there would likely be an extra compartment if detritus was separated into different taxa. Second, there are different definitions for grouping species within food webs, such as trophic groups (i.e., groups of species that are functionally similar) and trophic modules or motifs (i.e., the basic building blocks of food webs, usually but not necessarily containing three nodes). Generally food webs contain a large number of trophic groups (Gauzens et al., 2015) or trophic modules (Bascompte, 2009; Kondoh, 2008). Third, the scale of the systems is different. Generally food webs have smaller sizes (e.g., 23–124 taxa in our study) than other networks. The statement raised by Simon (1962) refers to systems that are much larger with many different parts, and therefore, it is not surprising that the number of compartments found here is relatively small. In addition, food webs are treated as a weighted and directed graph, which means the compartments are restricted by the underlying tree structure due to the direction of energy flow. This might also contribute to the small number of compartments found.

Our algorithm based on energy channels generated either similar or higher values of modularity when compared with commonly used community detection algorithms in complex network studies, that is, algorithms based on edge betweenness (Newman & Girvan, 2004) and random walk (Pons & Latapy, 2005). This shows the high feasibility of our algorithm and the high compartmentalization of different channels, especially considering that the other two algorithms use optimization to maximize the value of modularity.

Based on our results, several pairs of energy channels could be regarded as the compartments of a food web, including pelagic versus benthic channels, macrophyte‐derived versus phytoplankton‐derived channels, bacteria‐derived versus algae‐derived channels, and terrestrial (i.e., leaf/wood‐derived) versus aquatic channels. This is not in conflict with the argument that compartments arise from subhabitats (Krause et al., 2003; Pimm, 1991). Each habitat requires a set of adaptations from its component species, which may preclude a large number of interactions between species in different habitats and encourage more within‐habitat interactions (Pimm, 1991), that is, creating habitat‐based compartments. Meanwhile, different habitats can sustain different energy channels, for example, pelagic versus benthic and terrestrial versus aquatic. Moreover, there can still be different compartments within the same subhabitat, for example, benthic bacteria and benthic phytoplankton, and sometimes different subhabitats may share the same energy channel, depending on the scale of subhabitats. This suggests that energy channels and subhabitats may both contribute to the compartmentalization of food webs. Besides, our results appear to emphasize the physical separation between benthic and pelagic habitats. This implies the limitation of our datasets: All the 28 food webs we studied are aquatic or at least contain aquatic part. This to some extent limits the application of our conclusions, although it would be interesting to apply our algorithm to terrestrial or other food webs and compare the results with our conclusions in this study.

Basal resources (producers and detritus) often exhibit quite variable traits, such as habitat type and body size, which can attract very different types of primary consumers (Rooney & McCann, 2012). This characteristic supports diverse energy channels, which are in turn coupled by mobile predators at higher trophic levels. The partition of food webs in our study suggests that this coupling is not so strong and that the channels can be highly independent of one another. Therefore, potential perturbations should be constrained within a single compartment, as suggested in previous studies (Krause et al., 2003; Stouffer & Bascompte, 2011). This may be a vital stabilizing characteristic of natural ecosystems in the face of accelerating biodiversity loss (Barnosky et al., 2011).

## CONFLICT OF INTERESTS

We have no competing interests.

## DATA ACCESSIBILITY

All data used in this paper are available in the “enaR” package (Borrett & Lau, 2014) in R (https://cran.r-project.org/web/packages/enaR/index.html).

## AUTHORS’ CONTRIBUTIONS

HZ and LZ were responsible for research design. LZ, XL and WT performed numerical simulation and statistical analyses. LZ drafted the main text and prepared the figures. All authors were involved in discussions and editing.
