# Supplementary material for: Identifying compartments in ecological networks based on energy channels
Source: Ecol Evol. 2017 Nov 28;8(1):309–18. doi: 10.1002/ece3.3648 (PMC5756831; doi:10.1002/ece3.3648)
Supplement: Supplementary file 1 [file ECE3-8-309-s001.docx]

**Supporting Information:**

**Identifying compartments in ecological networks based on energy channels**

**Lei Zhao^a, b, c^, Huayong Zhang^* a^, Wang Tian^a^, Xiang Xu^a^**

*^a^* *Research Center for Engineering Ecology and Nonlinear Science, North China Electric Power University, Beijing, 102206, China*

*^b^ Department of Ecology and Evolutionary Biology and Kansas Biological Survey, University of Kansas, Lawrence, Kansas 66047, USA*

*^c^ Department of Life Sciences, Imperial College London, Silwood Park Campus, Buckhurst Road, Ascot, Berkshire, SL5 7PY, UK*

**^*^ Corresponding author**: Huayong Zhang, Email: [rceens@ncepu.edu.cn](mailto:rceens@ncepu.edu.cn)

**Appendix S1** Setting and calculation of the parameters.

**Table S1** Details of the parameters used in the model.

| **Symbol** | **Meaning** | **Value** | **Unit** |
| --- | --- | --- | --- |
| *r_i_* | Maximum specific or intrinsic growth rate | $(GPP_{i}-R_{i})/\left[B_{i}(1-1/10^{k_{0}})\right]$ | day^-1^ |
| *K* | Carrying capacity | $10^{k_{0}}\sum_{j=pro} B_{i}$ | g C m^-2^ |
| *d_i_* | Natural specific death rate | $\sum_{j=\det} F_{ij}/B_{i}$ | day^-1^ |
| *a_i_* | Assimilation efficiency | $1-\sum_{j=\det} F_{ij}/\sum_{j=res} F_{ji}$ | proportion (unitless) |
| *x_i_* | Respiration rate | *R_i_* / *B_i_* | day^-1^ |
| *p_ji_* | Proportion of converted detritus *i* in all the converted detritus from producer or consumer taxon *j* | $F_{ji}/\sum_{k=\det} F_{jk}$ | proportion (unitless) |
| *e_i_* | Egestion rate | $1-a_{i}$ | proportion (unitless) |
| *c_ji_* | Conversion coefficient from detritus *j* to detritus *i* | *F_ji_* / *B_j_* | day^-1^ |

Our data (see Table 1) contain the values of *GPP_i_* (gross primary production), *R_i_* (respiration), *B_i_* (biomass), and *F_ij_* (carbon flux when taxon *j* consumes taxon *i*). The term in Eq. 10 $r_{i}B_{i}(1-\sum_{j=pro} B_{j}/K)$ describes the net growth of producer biomass per unit time, i.e. the net primary production (*NPP*). Since *NPP* equals the gross primary production (*GPP*) minus the respiration (*R*) of producers, we can get $r_{i}B_{i}(1-\sum_{j=pro} B_{j}/K)=GPP_{i}-R_{i}$. Thus, *r* can be calculated by $\frac{(GPP_{i}-R_{i})}{B_{i}(1-\sum_{j=res} B_{j}/K)}$. *k_0_* is an undetermined parameter. Considering that carrying capacity *K* was within three orders of magnitude of total primary producer biomass in the community being simulated (Hudson & Reuman 2013), we assumed *k_0_* follows the distribution U[0, 3]. We ran 1,000 separate simulations for each food web, using different values of *k_0_*, chosen randomly from this distribution.

The functional response *Φ_ij_* was set to follow either a nonlinear form or a linear form. The nonlinear form was set as follows (see Hudson & Reuman 2013):

$\Phi_{ij}=\frac{y_{j}\omega_{ij}B_{i}^{h}}{H_{j}^{h}+q_{j}B_{j}H_{j}^{h}+\sum_{k=res} \omega_{kj}B_{k}^{h}}$ (12)

Here *y_j_* is the maximum consumption rate of taxon *j* and *ω_ij_* is the preference of taxon *j* for taxon *i*. For a consumer *j*, $\omega_{ij}\propto F_{ij}/B_{i}^{h}$. *F_ij_* is the carbon flux from taxon *i* to taxon *j*, which was contained in the empirical data. Given that $\sum_{i=res} \omega_{ij}=1$, we can calculate *ω_ij_* as:

$\omega_{ij}=\frac{F_{ij}/B_{i}^{h}}{\sum_{k=res} F_{kj}/B_{k}^{h}}$ (13)

*H_j_* is the half-saturation density, which was one order of magnitude either side of the mean of all biomasses in the community being simulated (Hudson & Reuman 2013). That means$H_{j}=10^{b}\overline{B}$. Here *b* is a coefficient following the distribution U[-1, 1]. *q_j_* is the predator interference coefficient, which was randomly chosen from 0 to 100 (Hudson & Reuman 2013). *h* is the hill exponent that regulates the shape of the curve from Holling type II (*h* = 1) to Holling type III (*h* = 2). We chose the value of *h* randomly from 1 to 2.

Notice that $F_{ij}=\Phi_{ij}B_{j}$, combining Eq. 12, and we can calculate the value of *y_j_* by:

$y_{j}=\frac{F_{ij}(H_{j}^{h}+q_{j}B_{j}H_{j}^{h}+\sum_{k=res} \omega_{kj}B_{k}^{h})}{\omega_{ij}B_{i}^{h}B_{j}}$ (14)

We ran 1,000 simulations for each food web. The values of parameter *b*, *q*, and *h* for each simulation were chosen randomly from their ranges, i.e. U[-1, 1] for *b*, U[0, 100] for *q*, and U[1, 2] for *h*.

**Appendix S2** Compartments detected using our algorithm within all the food webs, except for the first one Chesapeake Bay which is demonstrated in Figure 2. Different colours indicate different compartments.

**2. Cypress (dry)**


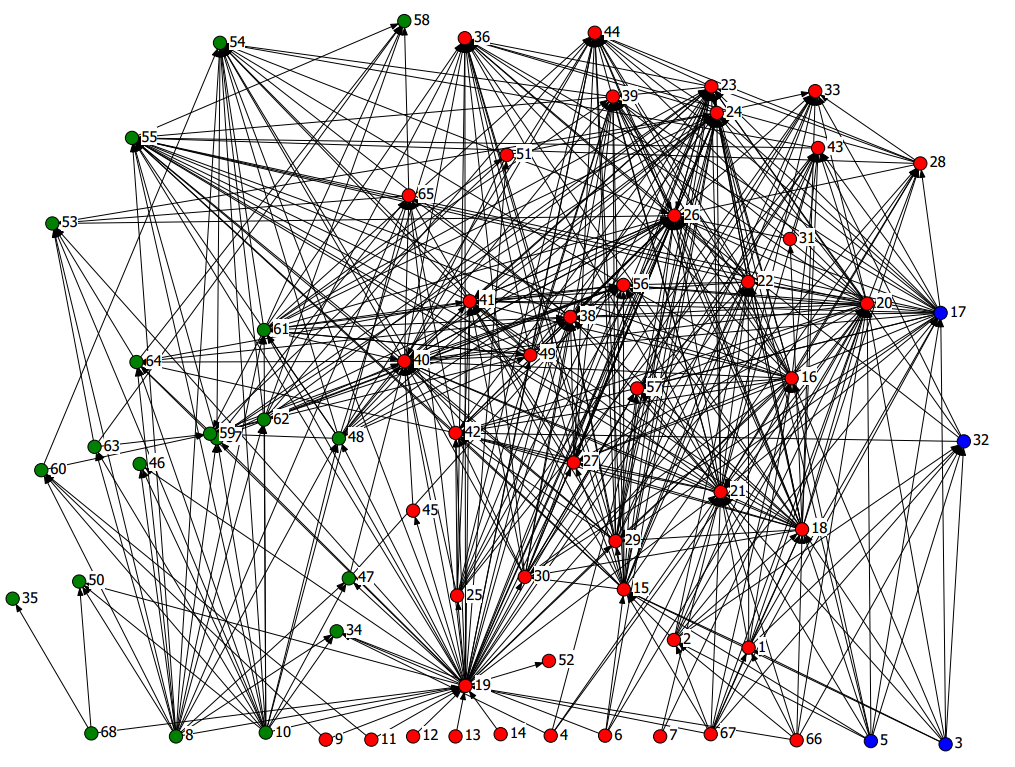


| **ID** | **Name** | **ID** | **Name** | **ID** | **Name** |
| --- | --- | --- | --- | --- | --- |
| **1** | **Living POC** | **24** | **Turtles** | **47** | **Woodpeckers** |
| **2** | **Living sediment** | **25** | **Lizards** | **48** | **Passeriformes onniv.** |
| **3** | **Phytoplankton** | **26** | **Snakes** | **49** | **Passeriformes pred.** |
| **4** | **Float. Vegetation** | **27** | **Salamanders** | **50** | **Opossum** |
| **5** | **Periphyton/Macroalgae** | **28** | **Large Frogs** | **51** | **Shrews** |
| **6** | **Macrophytes** | **29** | **Medium Frogs** | **52** | **Bats** |
| **7** | **Epiphytes** | **30** | **Small Frogs** | **53** | **Black Bear** |
| **8** | **Understory** | **31** | **Salamander L** | **54** | **G. Fox** |
| **9** | **Vine Leaves** | **32** | **Tadpoles** | **55** | **Raccoon** |
| **10** | **Hardwoods Leaves** | **33** | **Pelecaniformes** | **56** | **Mink** |
| **11** | **Cypress Leaves** | **34** | **Anseriformes** | **57** | **Otter** |
| **12** | **Cypress Wood** | **35** | **Vultures** | **58** | **Florida Panther** |
| **13** | **HW Wood** | **36** | **Kites & Hawks** | **59** | **Bobcat** |
| **14** | **Roots** | **37** | **Galliformes** | **60** | **Squirrels** |
| **15** | **Crayfish** | **38** | **Egrets** | **61** | **Mice & Rats** |
| **16** | **Apple Snail** | **39** | **Great blue heron** | **62** | **Rabbits** |
| **17** | **Prawn** | **40** | **Other herons** | **63** | **White-Tailed Deer** |
| **18** | **Aquatic Invertebrates** | **41** | **Wood stork** | **64** | **Hogs** |
| **19** | **Ter. Invertebrates** | **42** | **White ibis** | **65** | **Armadillo** |
| **20** | **Small Fish+herb+omniv** | **43** | **Gruiformes** | **66** | **Refractory Det.** |
| **21** | **Small Fish+prim.carniv** | **44** | **Owls** | **67** | **Liable Det.** |
| **22** | **Large Fish** | **45** | **Caprimulgiformes** | **68** | **Vertebrate Det.** |
| **23** | **Alligators** | **46** | **Hummingbirds** |  |  |

**3.** **Cypress (wet)**


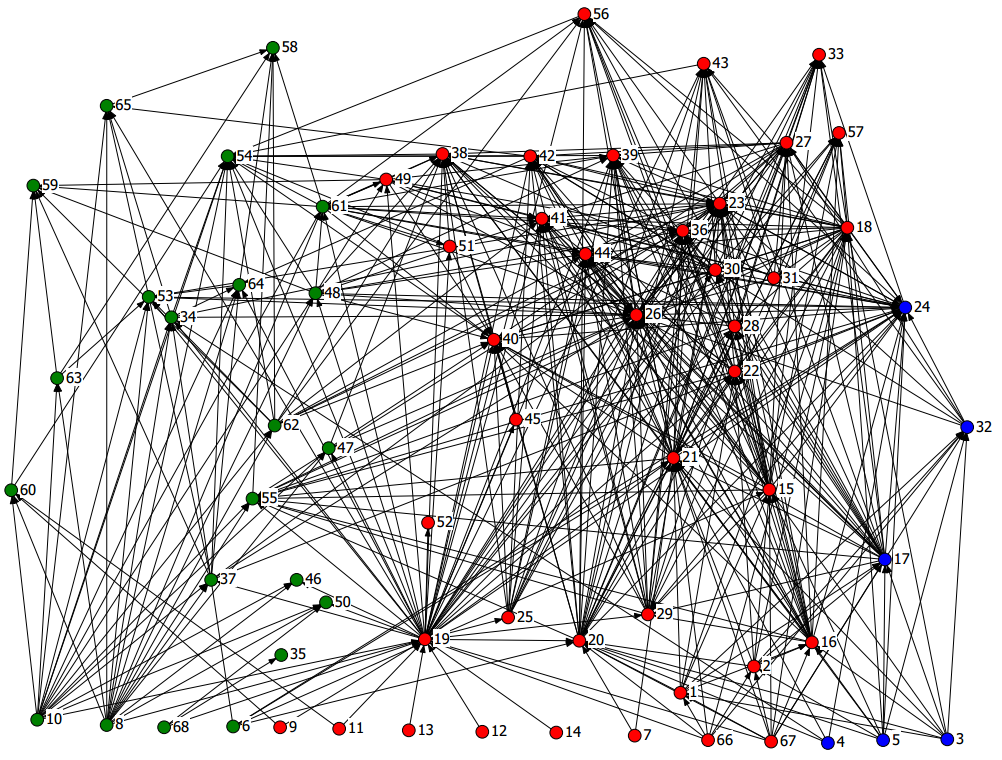


| **ID** | **Name** | **ID** | **Name** | **ID** | **Name** |
| --- | --- | --- | --- | --- | --- |
| **1** | **Living POC** | **24** | **Turtles** | **47** | **Woodpeckers** |
| **2** | **Living SED** | **25** | **Lizards** | **48** | **Passeriformes onniv.** |
| **3** | **Phytoplankton** | **26** | **Snakes** | **49** | **Passeriformes pred.** |
| **4** | **Float Veg.** | **27** | **Salamanders** | **50** | **Opossum** |
| **5** | **Periphyton** | **28** | **L Frog** | **51** | **Shrews** |
| **6** | **Macrophytes** | **29** | **M Frog** | **52** | **Bats** |
| **7** | **Epiphytes** | **30** | **S Frog** | **53** | **Black Bear** |
| **8** | **Understory** | **31** | **Salam. L** | **54** | **G Fox** |
| **9** | **Vine L** | **32** | **Tadpoles** | **55** | **Raccoon** |
| **10** | **Hardwood L** | **33** | **Pelecaniformes** | **56** | **Mink** |
| **11** | **Cypress L** | **34** | **Anseriformes** | **57** | **Otter** |
| **12** | **Cypress W** | **35** | **Vultures** | **58** | **Florida Panther** |
| **13** | **Hardwood W** | **36** | **Kites & Hawks** | **59** | **Bobcat** |
| **14** | **Roots** | **37** | **Galliformes** | **60** | **Squirrels** |
| **15** | **Crayfish** | **38** | **Egrets** | **61** | **Mice & Rats** |
| **16** | **Apple Snail** | **39** | **GB Heron** | **62** | **Rabbits** |
| **17** | **Prawn** | **40** | **Other Herons** | **63** | **White-Tailed Deer** |
| **18** | **Aquatic I** | **41** | **Wood stork** | **64** | **Hogs** |
| **19** | **Terrst. I** | **42** | **White ibis** | **65** | **Armadillo** |
| **20** | **Fish HO** | **43** | **Gruiformes** | **66** | **Refractory Det.** |
| **21** | **Fish PC** | **44** | **Owls** | **67** | **Liable Det.** |
| **22** | **L Fish** | **45** | **Caprimulgiformes** | **68** | **Vert. det** |
| **23** | **Alligators** | **46** | **Hummingbirds** |  |  |

**4.** **Florida Bay (dry)**


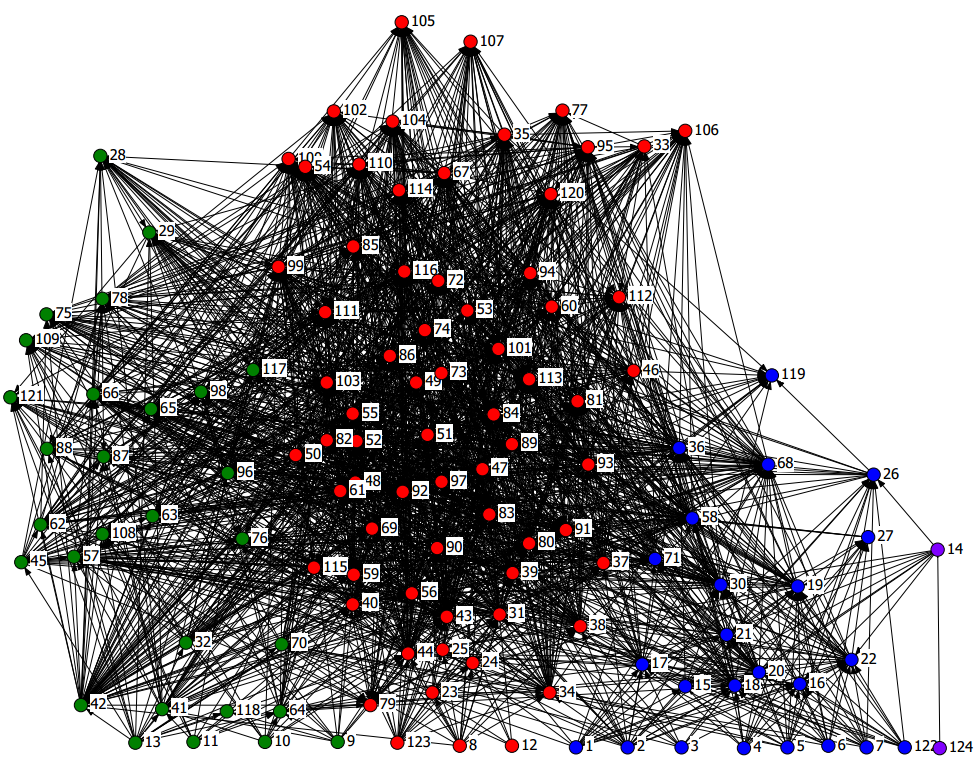


| **ID** | **Name** | **ID** | **Name** | **ID** | **Name** |
| --- | --- | --- | --- | --- | --- |
| **1** | **2um Spherical Phytoplankton** | **43** | **Herbivorous Shrimp** | **85** | **Scianids** |
| **2** | **Synedococcus** | **44** | **Pink Shrimp** | **86** | **Red Drum** |
| **3** | **Oscillatoria** | **45** | **Thor Floridanus** | **87** | **Spadefish** |
| **4** | **Small Diatoms (<20um)** | **46** | **Lobster** | **88** | **Parrotfish** |
| **5** | **Big Diatoms (>20um)** | **47** | **Detritivorous Crabs** | **89** | **Mackerel** |
| **6** | **Dinoflagellates** | **48** | **Omnivorous Crabs** | **90** | **Mullet** |
| **7** | **Other Phytoplankton** | **49** | **Predatory Crabs** | **91** | **Barracuda** |
| **8** | **Benthic Phytoplankton** | **50** | **Callinectus sapidus** | **92** | **Blennies** |
| **9** | **Thalassia** | **51** | **Stone Crab** | **93** | **Code Goby** |
| **10** | **Halodule** | **52** | **Sharks** | **94** | **Clown Goby** |
| **11** | **Syringodium** | **53** | **Rays** | **95** | **Flatfish** |
| **12** | **Drift Algae** | **54** | **Tarpon** | **96** | **Filefishes** |
| **13** | **Epiphytes** | **55** | **Bonefish** | **97** | **Puffer** |
| **14** | **Free Bacteria** | **56** | **Sardines** | **98** | **Other Pelagic Fishes** |
| **15** | **Water Flagellates** | **57** | **Anchovy** | **99** | **Other Demersal Fishes** |
| **16** | **Water Cilitaes** | **58** | **Bay Anchovy** | **100** | **Loon** |
| **17** | **Acartia Tonsa** | **59** | **Lizardfish** | **101** | **Greeb** |
| **18** | **Oithona nana** | **60** | **Catfish** | **102** | **Pelican** |
| **19** | **Paracalanus** | **61** | **Eels** | **103** | **Comorant** |
| **20** | **Other Copepoda** | **62** | **Toadfish** | **104** | **Big Herons & Egrets** |
| **21** | **Meroplankton** | **63** | **Brotalus** | **105** | **Small Herons & Egrets** |
| **22** | **Other Zooplankton** | **64** | **Halfbeaks** | **106** | **Ibis** |
| **23** | **Benthic Flagellates** | **65** | **Needlefish** | **107** | **Roseate Spoonbill** |
| **24** | **Benthic Ciliates** | **66** | **Other Killifish** | **108** | **Herbivorous Ducks** |
| **25** | **Meiofauna** | **67** | **Goldspotted killifish** | **109** | **Omnivorous Ducks** |
| **26** | **Sponges** | **68** | **Rainwater killifish** | **110** | **Predatory Ducks** |
| **27** | **Coral** | **69** | **Snook** | **111** | **Raptors** |
| **28** | **Other Cnidaridae** | **70** | **Sailfin Molly** | **112** | **Gruiformes** |
| **29** | **Echinoderma** | **71** | **Silverside** | **113** | **Small Shorebirds** |
| **30** | **Bivalves** | **72** | **Other Horsefish** | **114** | **Gulls & Terns** |
| **31** | **Detritivorous Gastropods** | **73** | **Gulf Pipefish** | **115** | **Kingfisher** |
| **32** | **Epiphytic Gastropods** | **74** | **Dwarf Seahorse** | **116** | **Crocodiles** |
| **33** | **Predatory Gastropods** | **75** | **Grouper** | **117** | **Loggerhead Turtle** |
| **34** | **Detritivorous Polychaetes** | **76** | **Jacks** | **118** | **Green Turtle** |
| **35** | **Predatory Polychaetes** | **77** | **Pompano** | **119** | **Hawksbill Turtle** |
| **36** | **Suspension Feeding Polych** | **78** | **Other Snapper** | **120** | **Dolphin** |
| **37** | **Macrobenthos** | **79** | **Gray Snapper** | **121** | **Manatee** |
| **38** | **Benthic Crustaceans** | **80** | **Mojarra** | **122** | **Water POC** |
| **39** | **Detritivorous Amphipods** | **81** | **Grunt** | **123** | **Benthic POC** |
| **40** | **Herbivorous Amphipods** | **82** | **Porgy** | **124** | **DOC** |
| **41** | **Isopods** | **83** | **Pinfish** |  |  |
| **42** | **Predatory Shrimp** | **84** | **Spotted Seatrout** |  |  |

**5.** **Florida Bay (wet)**


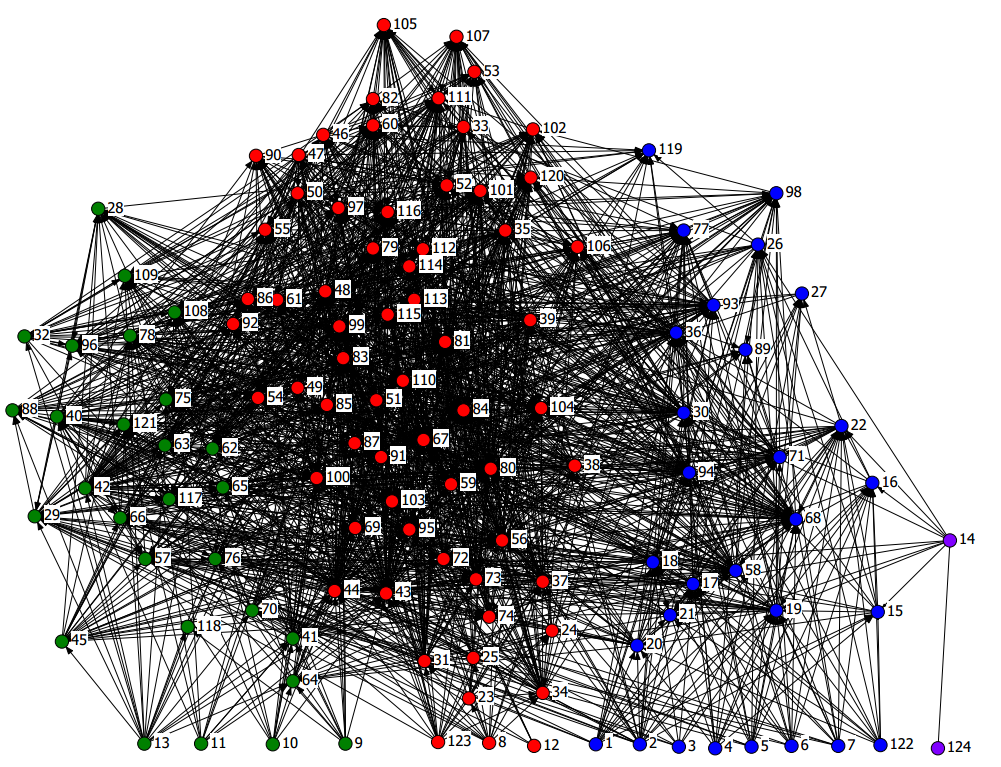


| **ID** | **Name** | **ID** | **Name** | **ID** | **Name** |
| --- | --- | --- | --- | --- | --- |
| **1** | **2um Spherical Phytoplankton** | **43** | **Predatory Shrimp** | **85** | **Spotted Seatrout** |
| **2** | **Synedococcus** | **44** | **Pink Shrimp** | **86** | **Red Drum** |
| **3** | **Oscillatoria** | **45** | **Thor Floridanus** | **87** | **Spadefish** |
| **4** | **Small Diatoms (<20um)** | **46** | **Lobster** | **88** | **Parrotfish** |
| **5** | **Big Diatoms (>20um)** | **47** | **Detritivorous Crabs** | **89** | **Mackerel** |
| **6** | **Dinoflagellates** | **48** | **Omnivorous Crabs** | **90** | **Mullet** |
| **7** | **Other Phytoplankton** | **49** | **Predatory Crabs** | **91** | **Barracuda** |
| **8** | **Benthic Phytoplankton** | **50** | **Callinectus sapidus** | **92** | **Blennies** |
| **9** | **Thalassia** | **51** | **Stone Crab** | **93** | **Code Goby** |
| **10** | **Halodule** | **52** | **Sharks** | **94** | **Clown Goby** |
| **11** | **Syringodium** | **53** | **Rays** | **95** | **Flatfish** |
| **12** | **Drift Algae** | **54** | **Tarpon** | **96** | **Filefishes** |
| **13** | **Epiphytes** | **55** | **Bonefish** | **97** | **Puffer** |
| **14** | **Free Bacteria** | **56** | **Sardines** | **98** | **Other Pelagic Fishes** |
| **15** | **Water Flagellates** | **57** | **Anchovy** | **99** | **Other Demersal Fishes** |
| **16** | **Water Cilitaes** | **58** | **Bay Anchovy** | **100** | **Loon** |
| **17** | **Acartia Tonsa** | **59** | **Lizardfish** | **101** | **Greeb** |
| **18** | **Oithona nana** | **60** | **Catfish** | **102** | **Pelican** |
| **19** | **Paracalanus** | **61** | **Eels** | **103** | **Comorant** |
| **20** | **Other Copepoda** | **62** | **Toadfish** | **104** | **Big Herons & Egrets** |
| **21** | **Meroplankton** | **63** | **Brotalus** | **105** | **Small Herons & Egrets** |
| **22** | **Other Zooplankton** | **64** | **Halfbeaks** | **106** | **Ibis** |
| **23** | **Benthic Flagellates** | **65** | **Needlefish** | **107** | **Roseate Spoonbill** |
| **24** | **Benthic Ciliates** | **66** | **Other Killifish** | **108** | **Herbivorous Ducks** |
| **25** | **Meiofauna** | **67** | **Goldspotted killifish** | **109** | **Omnivorous Ducks** |
| **26** | **Sponges** | **68** | **Rainwater killifish** | **110** | **Predatory Ducks** |
| **27** | **Coral** | **69** | **Snook** | **111** | **Raptors** |
| **28** | **Other Cnidaridae** | **70** | **Sailfin Molly** | **112** | **Gruiformes** |
| **29** | **Echinoderma** | **71** | **Silverside** | **113** | **Small Shorebirds** |
| **30** | **Bivalves** | **72** | **Other Horsefish** | **114** | **Gulls & Terns** |
| **31** | **Detritivorous Gastropods** | **73** | **Gulf Pipefish** | **115** | **Kingfisher** |
| **32** | **Epiphytic Gastropods** | **74** | **Dwarf Seahorse** | **116** | **Crocodiles** |
| **33** | **Predatory Gastropods** | **75** | **Grouper** | **117** | **Loggerhead Turtle** |
| **34** | **Detritivorous Polychaetes** | **76** | **Jacks** | **118** | **Green Turtle** |
| **35** | **Predatory Polychaetes** | **77** | **Pompano** | **119** | **Hawksbill Turtle** |
| **36** | **Suspension Feeding Polych** | **78** | **Other Snapper** | **120** | **Dolphin** |
| **37** | **Macrobenthos** | **79** | **Gray Snapper** | **121** | **Manatee** |
| **38** | **Benthic Crustaceans** | **80** | **Mojarra** | **122** | **Water POC** |
| **39** | **Detritivorous Amphipods** | **81** | **Grunt** | **123** | **Benthic POC** |
| **40** | **Herbivorous Amphipods** | **82** | **Porgy** | **124** | **DOC** |
| **41** | **Isopods** | **83** | **Pinfish** |  |  |
| **42** | **Herbivorous Shrimp** | **84** | **Scianids** |  |  |

**6.** **Georges Bank**


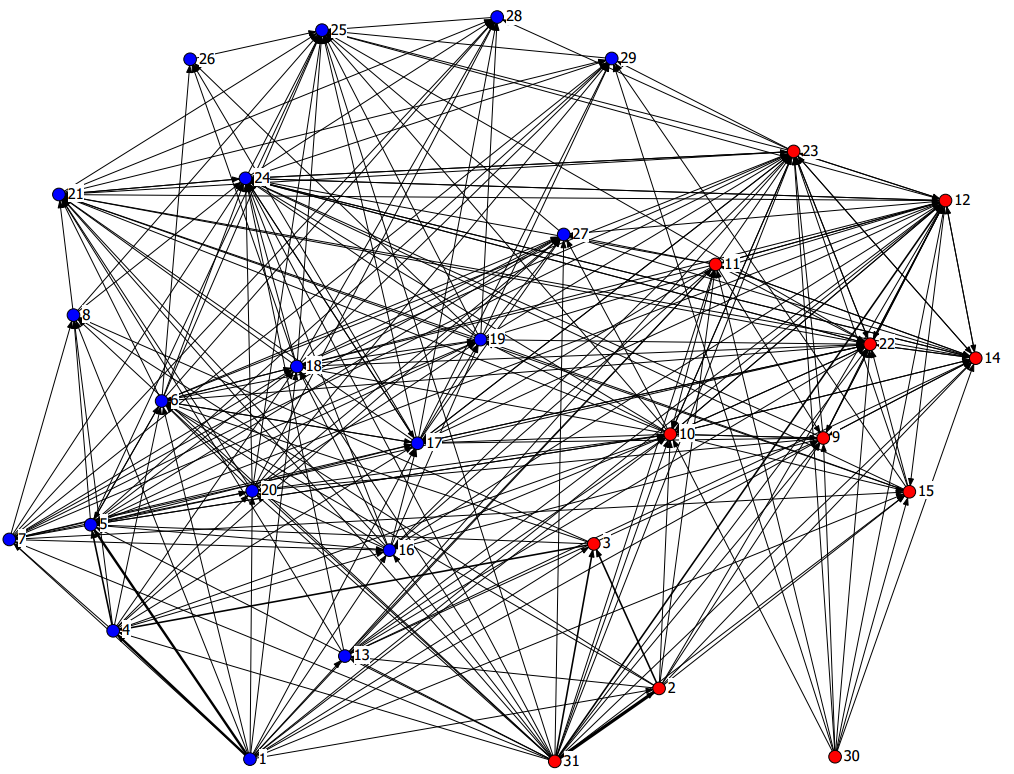


| **ID** | **Name** | **ID** | **Name** | **ID** | **Name** |
| --- | --- | --- | --- | --- | --- |
| **1** | **Phytoplankton- Primary** | **12** | **Macrobenthos- other** | **23** | **Demersals- omnivores** |
| **2** | **Bacteria** | **13** | **Megabenthos- filterers** | **24** | **Demersals- piscivores** |
| **3** | **Microzooplankton** | **14** | **Megabenthos- other** | **25** | **Sharks- pelagics** |
| **4** | **Small copepods** | **15** | **Shrimp et al.** | **26** | **Highly Migratory Speci** |
| **5** | **Large Copepods** | **16** | **Larval-juv fish- all** | **27** | **Baleen Whales** |
| **6** | **Gelatinous Zooplankton** | **17** | **Small Pelagics- commer** | **28** | **Odontocetes** |
| **7** | **Micronekton** | **18** | **Small Pelagics- other** | **29** | **Sea Birds** |
| **8** | **Mesopelagics** | **19** | **Small Pelagics- squid** | **30** | **Discards** |
| **9** | **Macrobenthos- polychaet** | **20** | **Small Pelagics- anadro** | **31** | **Detritus- POC** |
| **10** | **Macrobenthos- crustace** | **21** | **Medium Pelagics- pisc** |  |  |
| **11** | **Macrobenthos- molluscs** | **22** | **Demersals- benthivores** |  |  |

**7.** **Graminoids (dry)**


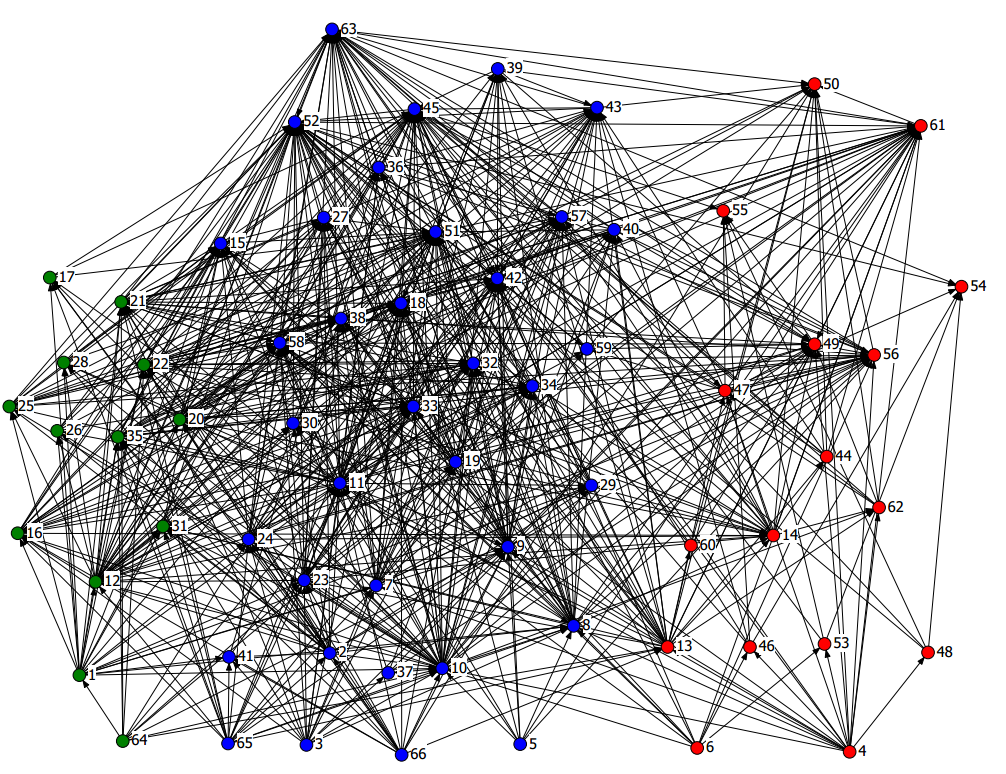


| **ID** | **Name** | **ID** | **Name** | **ID** | **Name** |
| --- | --- | --- | --- | --- | --- |
| **1** | **Living Sediments** | **23** | **Mosquitofishes** | **45** | **Alligators** |
| **2** | **Living POC** | **24** | **Poecilids** | **46** | **Muskrats** |
| **3** | **Periphyton** | **25** | **Pigmy Sunfish** | **47** | **Rats&Mice** |
| **4** | **Macrophytes** | **26** | **Bluespotted Sunfish** | **48** | **Rabbits** |
| **5** | **Utricularia** | **27** | **Warmouth** | **49** | **Raccoons** |
| **6** | **Floating Veg.** | **28** | **Dollar Sunfish** | **50** | **Opossum** |
| **7** | **Apple snail** | **29** | **Redear Sunfish** | **51** | **Otter** |
| **8** | **Freshwater Prawn** | **30** | **Spotted sunfish** | **52** | **Mink** |
| **9** | **Crayfish** | **31** | **Other Centrarchids** | **53** | **W-T Deer** |
| **10** | **Mesoinverts** | **32** | **Largemouth Bass** | **54** | **Bobcat** |
| **11** | **Other Macroinverts** | **33** | **Cichlids** | **55** | **Panthers** |
| **12** | **Large Aquatic Insects** | **34** | **Other Large Fishes** | **56** | **Grebes** |
| **13** | **Terrestrial Inverts** | **35** | **Other Small Fishes** | **57** | **Bitterns** |
| **14** | **Fishing spider** | **36** | **Salamanders** | **58** | **Ducks** |
| **15** | **Gar** | **37** | **Salamander larvae** | **59** | **Snailkites** |
| **16** | **Shiners & Minnows** | **38** | **Large frogs** | **60** | **Nighthawks** |
| **17** | **Chubsuckers** | **39** | **Medium frogs** | **61** | **Gruiformes** |
| **18** | **Catfish** | **40** | **Small frogs** | **62** | **CSSsparrow** |
| **19** | **Flagfish** | **41** | **Tadpoles** | **63** | **Passerines** |
| **20** | **Topminnows** | **42** | **Turtles** | **64** | **Sediment Carbon** |
| **21** | **Bluefin killifish** | **43** | **Snakes** | **65** | **Labile Detritus** |
| **22** | **Killifishes** | **44** | **Lizards** | **66** | **Refractory Detritus** |

**8.** **Graminoids (wet)**


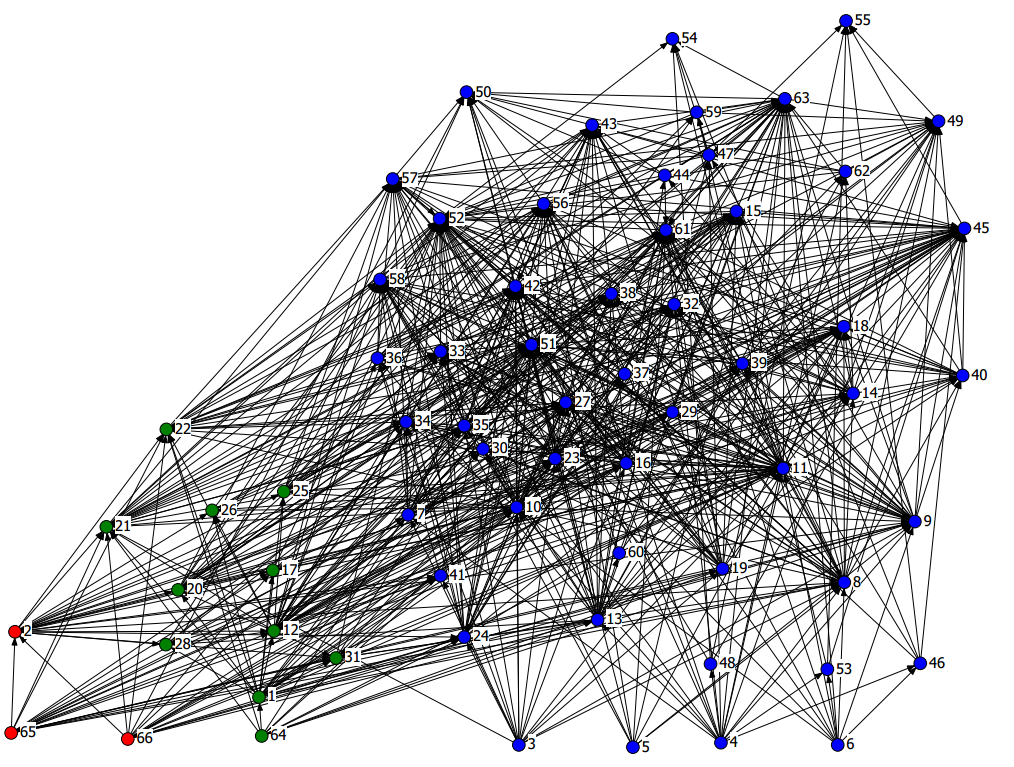


| **ID** | **Name** | **ID** | **Name** | **ID** | **Name** |
| --- | --- | --- | --- | --- | --- |
| **1** | **Living Sediments** | **23** | **Mosquitofishes** | **45** | **Alligators** |
| **2** | **Living POC** | **24** | **Poecilids** | **46** | **Muskrats** |
| **3** | **Periphyton** | **25** | **Pigmy Sunfish** | **47** | **Rats&Mice** |
| **4** | **Macrophytes** | **26** | **Bluespotted Sunfish** | **48** | **Rabbits** |
| **5** | **Utricularia** | **27** | **Warmouth** | **49** | **Raccoons** |
| **6** | **Floating Veg.** | **28** | **Dollar Sunfish** | **50** | **Opossum** |
| **7** | **Apple snail** | **29** | **Redear Sunfish** | **51** | **Otter** |
| **8** | **Freshwater Prawn** | **30** | **Spotted sunfish** | **52** | **Mink** |
| **9** | **Crayfish** | **31** | **Other Centrarchids** | **53** | **W-T Deer** |
| **10** | **Mesoinverts** | **32** | **Largemouth Bass** | **54** | **Bobcat** |
| **11** | **Other Macroinverts** | **33** | **Cichlids** | **55** | **Panthers** |
| **12** | **Large Aquatic Insects** | **34** | **Other Large Fishes** | **56** | **Grebes** |
| **13** | **Terrestrial Inverts** | **35** | **Other Small Fishes** | **57** | **Bitterns** |
| **14** | **Fishing spider** | **36** | **Salamanders** | **58** | **Ducks** |
| **15** | **Gar** | **37** | **Salamander larvae** | **59** | **Snailkites** |
| **16** | **Shiners & Minnows** | **38** | **Large frogs** | **60** | **Nighthawks** |
| **17** | **Chubsuckers** | **39** | **Medium frogs** | **61** | **Gruiformes** |
| **18** | **Catfish** | **40** | **Small frogs** | **62** | **CSSsparrow** |
| **19** | **Flagfish** | **41** | **Tadpoles** | **63** | **Passerines** |
| **20** | **Topminnows** | **42** | **Turtles** | **64** | **Sediment Carbon** |
| **21** | **Bluefin killifish** | **43** | **Snakes** | **65** | **Labile Detritus** |
| **22** | **Killifishes** | **44** | **Lizards** | **66** | **Refractory Detritus** |

**9.** **Gulf of Maine**


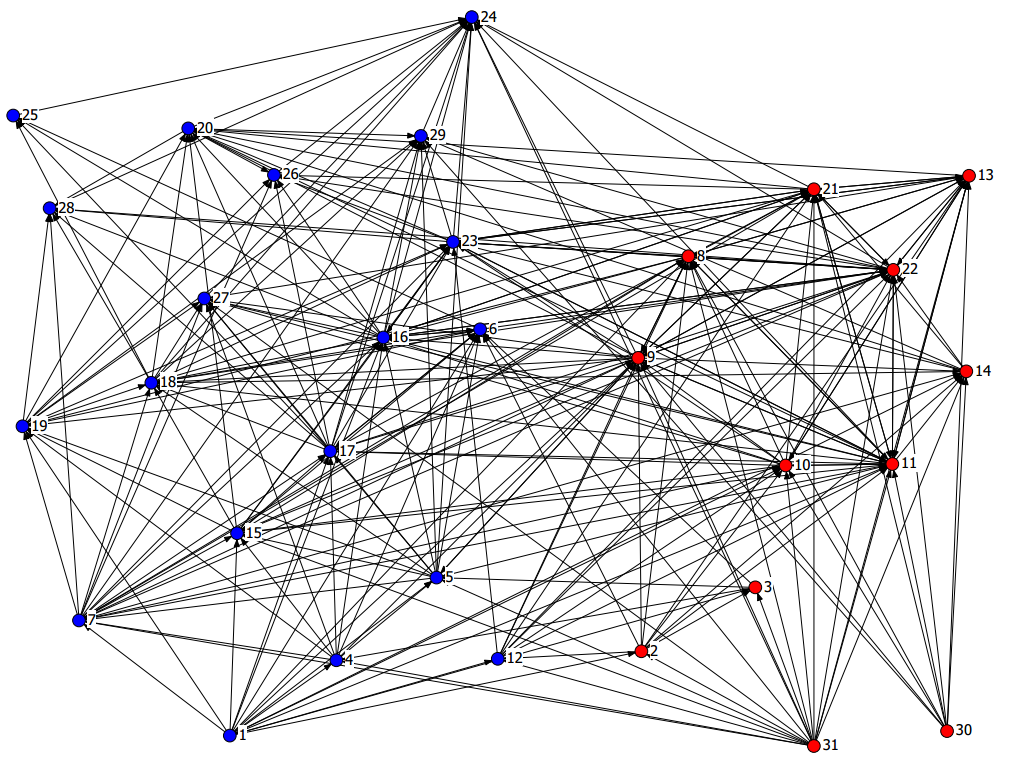


| **ID** | **Name** | **ID** | **Name** | **ID** | **Name** |
| --- | --- | --- | --- | --- | --- |
| **1** | **Phytoplankton- Primary** | **12** | **Megabenthos- filterers** | **23** | **Demersals- piscivores** |
| **2** | **Bacteria** | **13** | **Megabenthos- other** | **24** | **Sharks- pelagics** |
| **3** | **Microzooplankton** | **14** | **Shrimp et al.** | **25** | **Highly Migratory Speci** |
| **4** | **Small copepods** | **15** | **Larval-juv fish- all** | **26** | **Pinnipeds** |
| **5** | **Large Copepods** | **16** | **Small Pelagics- commer** | **27** | **Baleen Whales** |
| **6** | **Gelatinous Zooplankton** | **17** | **Small Pelagics- other** | **28** | **Odontocetes** |
| **7** | **Micronekton** | **18** | **Small Pelagics- squid** | **29** | **Sea Birds** |
| **8** | **Macrobenthos- polychaet** | **19** | **Small Pelagics- anadro** | **30** | **Discards** |
| **9** | **Macrobenthos- crustacea** | **20** | **Medium Pelagics- (pisc** | **31** | **Detritus- POC** |
| **10** | **Macrobenthos- molluscs** | **21** | **Demersals- benthivores** |  |  |
| **11** | **Macrobenthos- other** | **22** | **Demersals- omnivores** |  |  |

**10.** **Lake Oneida (pre-ZM)**


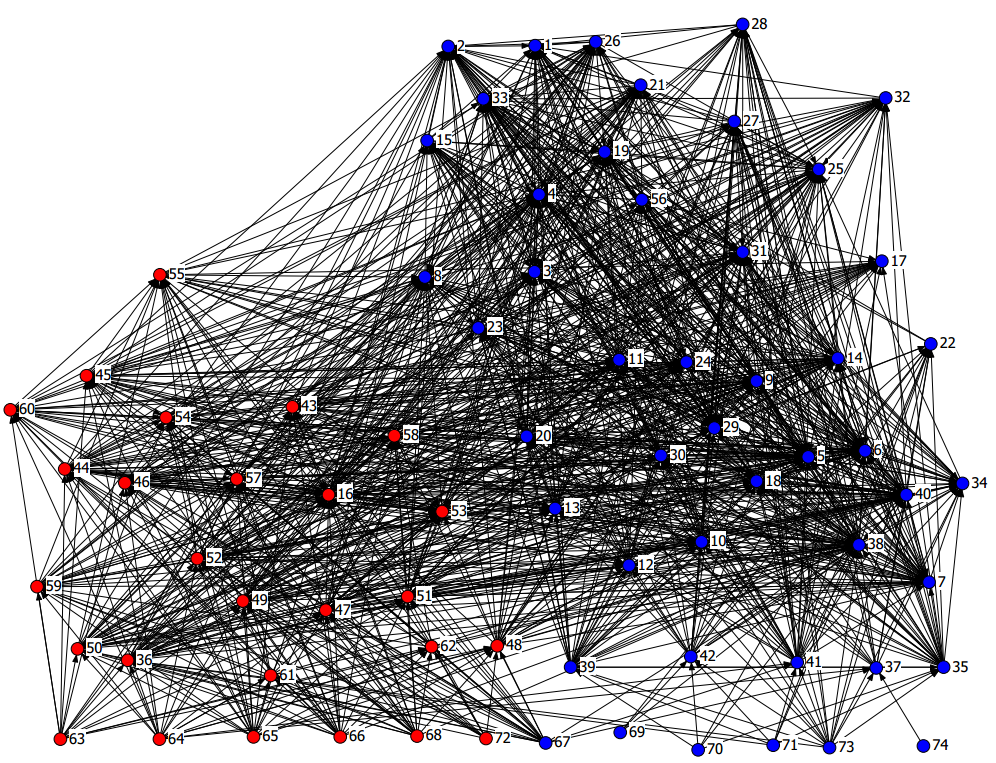


| **ID** | **Name** | **ID** | **Name** | **ID** | **Name** |
| --- | --- | --- | --- | --- | --- |
| **1** | **Cormorants** | **26** | **Golden Shiners** | **51** | **Eubosmina coregoni** |
| **2** | **Walleye Age 4+** | **27** | **Log Perch** | **52** | **Sida crystallina** |
| **3** | **Walleye Age 1-3** | **28** | **Mottled Sculpin** | **53** | **Leptodora kindtii** |
| **4** | **Walleye Age 0** | **29** | **Northern Pike** | **54** | **Acanthocyclops vernalis** |
| **5** | **Yellow Perch Age 3+** | **30** | **Red Horse Sucker** | **55** | **Diacyclops thomasi** |
| **6** | **Yellow Perch Age 1-2** | **31** | **Smallmouth Bass** | **56** | **Ergasilus species** |
| **7** | **Yellow Perch Age 0** | **32** | **Trout-perch** | **57** | **Mesocyclops edax** |
| **8** | **White Perch Age 1+** | **33** | **White Bass** | **58** | **Epischura lacustris** |
| **9** | **White Perch Age 0** | **34** | **White Sucker** | **59** | **Leptodiaptomus minutus** |
| **10** | **Black Crappie Age 1+** | **35** | **Amphipods** | **60** | **Skistodiaptomus oregonens** |
| **11** | **Bluegill Age 1+** | **36** | **Chironomids** | **61** | **Nauplii** |
| **12** | **Pumpkinseed Age 1+** | **37** | **Clams** | **62** | **Rotifers** |
| **13** | **Rock Bass Age 1+** | **38** | **Insects** | **63** | **Blue-green Algae** |
| **14** | **Panfish Age 0** | **39** | **Isopods** | **64** | **Diatoms** |
| **15** | **Gizzard Shad Age 1+** | **40** | **Leeches** | **65** | **Euglena** |
| **16** | **Gizzard Shad Age 0** | **41** | **Oligochaetes** | **66** | **Flagellates** |
| **17** | **Alewife** | **42** | **Snails** | **67** | **Golden Algae** |
| **18** | **Brown Bullhead** | **43** | **Alona species** | **68** | **Green Algae** |
| **19** | **Burbot** | **44** | **Bosmina longirostris** | **69** | **Epiphytes** |
| **20** | **Channel Catfish** | **45** | **Ceriodaphnia quadrangula** | **70** | **Macrophytes** |
| **21** | **Cisco** | **46** | **Chydorus sphaericus** | **71** | **Periphytes** |
| **22** | **Common Carp** | **47** | **Daphnia galeata mendotae** | **72** | **Pelagic Detritus** |
| **23** | **Darters** | **48** | **Daphnia pulicaria** | **73** | **Sedimented Detritus** |
| **24** | **Emerald Shiners** | **49** | **Daphnia retrocurva** | **74** | **DOC** |
| **25** | **Freshwater Drum** | **50** | **Diaphanosoma species** |  |  |

**11.** **Lake Oneida (post-ZM)**


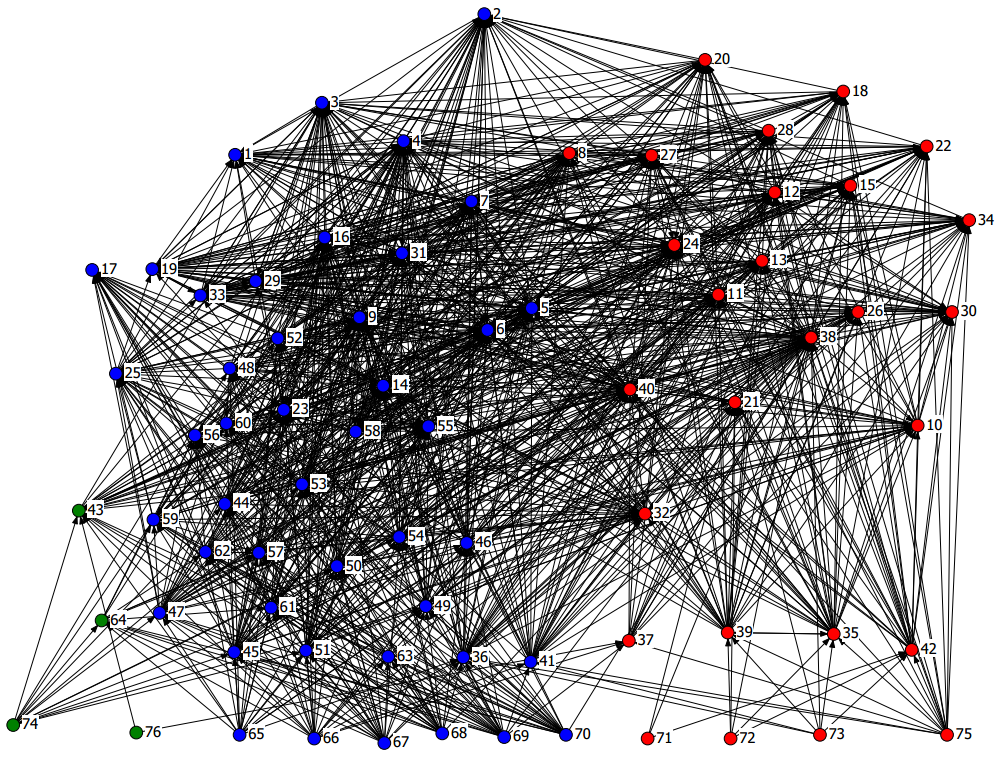


| **ID** | **Name** | **ID** | **Name** | **ID** | **Name** |
| --- | --- | --- | --- | --- | --- |
| **1** | **Cormorants** | **27** | **Log Perch** | **53** | **Eubosmina coregoni** |
| **2** | **Walleye Age 4+** | **28** | **Mottled Sculpin** | **54** | **Sida crystallina** |
| **3** | **Walleye Age 1-3** | **29** | **Northern Pike** | **55** | **Leptodora kindtii** |
| **4** | **Walleye Age 0** | **30** | **Red Horse Sucker** | **56** | **Acanthocyclops vernalis** |
| **5** | **Yellow Perch Age 3+** | **31** | **Smallmouth Bass** | **57** | **Diacyclops thomasi** |
| **6** | **Yellow Perch Age 1-2** | **32** | **Trout-perch** | **58** | **Ergasilus species** |
| **7** | **Yellow Perch Age 0** | **33** | **White Bass** | **59** | **Mesocyclops edax** |
| **8** | **White Perch Age 1+** | **34** | **White Sucker** | **60** | **Epischura lacustris** |
| **9** | **White Perch Age 0** | **35** | **Amphipods** | **61** | **Leptodiaptomus minutus** |
| **10** | **Black Crappie Age 1+** | **36** | **Chironomids** | **62** | **Skistodiaptomus oregonens** |
| **11** | **Bluegill Age 1+** | **37** | **Clams** | **63** | **Nauplii** |
| **12** | **Pumpkinseed Age 1+** | **38** | **Insects** | **64** | **Rotifers** |
| **13** | **Rock Bass Age 1+** | **39** | **Isopods** | **65** | **Blue-green Algae** |
| **14** | **Panfish Age 0** | **40** | **Leeches** | **66** | **Diatoms** |
| **15** | **Gizzard Shad Age 1+** | **41** | **Oligochaetes** | **67** | **Euglena** |
| **16** | **Gizzard Shad Age 0** | **42** | **Snails** | **68** | **Flagellates** |
| **17** | **Alewife** | **43** | **Zebra Mussels** | **69** | **Golden Algae** |
| **18** | **Brown Bullhead** | **44** | **Alona species** | **70** | **Green Algae** |
| **19** | **Burbot** | **45** | **Bosmina longirostris** | **71** | **Epiphytes** |
| **20** | **Channel Catfish** | **46** | **Camptocercus harpae** | **72** | **Macrophytes** |
| **21** | **Common Carp** | **47** | **Ceriodaphnia quadrangula** | **73** | **Periphytes** |
| **22** | **Darters** | **48** | **Chydorus sphaericus** | **74** | **Pelagic Detritus** |
| **23** | **Emerald Shiners** | **49** | **Daphnia galeata mendotae** | **75** | **Sedimented Detritus** |
| **24** | **Freshwater Drum** | **50** | **Daphnia pulicaria** | **76** | **DOC** |
| **25** | **Golden Shiners** | **51** | **Daphnia retrocurva** |  |  |
| **26** | **Lake Sturgeon** | **52** | **Diaphanosoma species** |  |  |

**12.** **Mangroves (dry)**


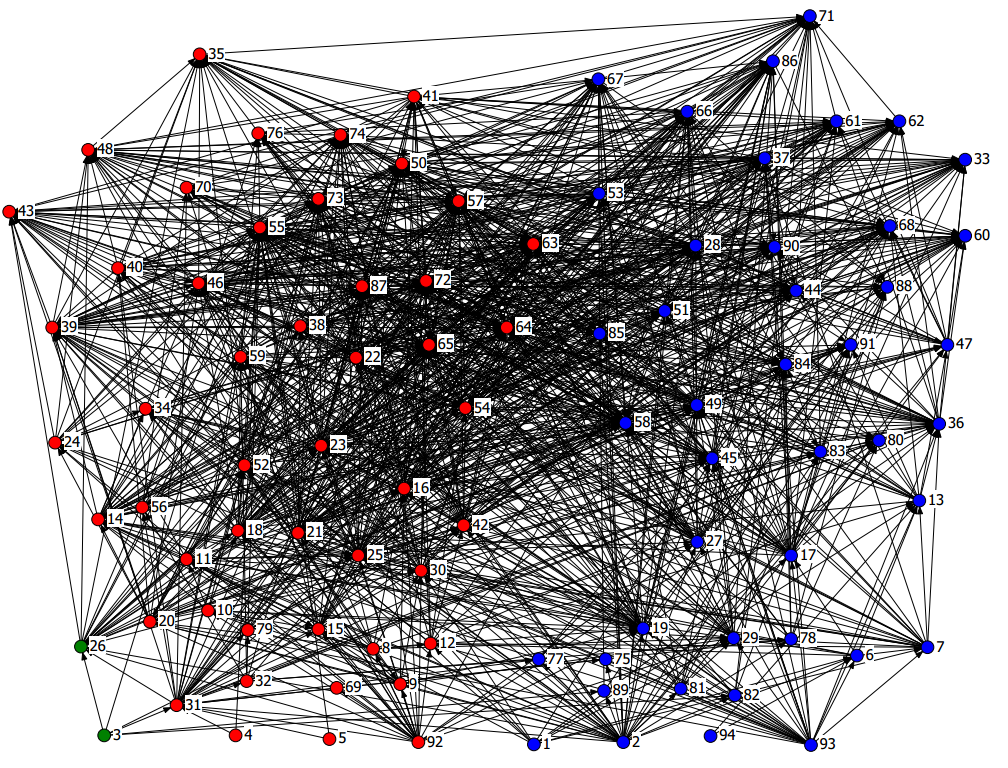


| **ID** | **Name** | **ID** | **Name** | **ID** | **Name** | **ID** | **Name** | **ID** | **Name** |
| --- | --- | --- | --- | --- | --- | --- | --- | --- | --- |
| **1** | **PHY** | **21** | **PENAID** | **41** | **HRSE** | **61** | **PELC** | **81** | **RABT** |
| **2** | **OTH. PP** | **22** | **CARID** | **42** | **SLVR** | **62** | **CORM** | **82** | **SQIUR** |
| **3** | **LEAF** | **23** | **OSHMP** | **43** | **SNOOK** | **63** | **BH & E** | **83** | **M & R** |
| **4** | **WOOD** | **24** | **JLOBST** | **44** | **CUDA** | **64** | **SE & E** | **84** | **FOX** |
| **5** | **ROOT** | **25** | **OCRAB** | **45** | **FWFSH** | **65** | **IBIS** | **85** | **BEAR** |
| **6** | **MICR. H2O** | **26** | **TCRAB** | **46** | **BENTH** | **66** | **DUCK1** | **86** | **RACO** |
| **7** | **ZOOPL.** | **27** | **DCRAB** | **47** | **EFISH** | **67** | **DUCK2** | **87** | **M & O** |
| **8** | **BACT.SED.** | **28** | **PCRAB** | **48** | **SNAP** | **68** | **DUCK3** | **88** | **CATS** |
| **9** | **FLA. SED.** | **29** | **FWINV** | **49** | **MOJA** | **69** | **VULT** | **89** | **DERS** |
| **10** | **CIL. SED.** | **30** | **LARV** | **50** | **SCIAE** | **70** | **K & H** | **90** | **DOLP** |
| **11** | **MEIOF.** | **31** | **INSCT** | **51** | **PIN** | **71** | **MRAPT** | **91** | **MANA** |
| **12** | **MERO** | **32** | **SPIDR** | **52** | **MULL** | **72** | **GUIF** | **92** | **C in SED** |
| **13** | **EPIFN** | **33** | **SHRK** | **53** | **GOBY** | **73** | **SSBIRDS** | **93** | **POC** |
| **14** | **POLY** | **34** | **RAYS** | **54** | **OFISH** | **74** | **G & T** | **94** | **DOC** |
| **15** | **TGAST** | **35** | **TARP** | **55** | **TURT** | **75** | **C & C** |  |  |
| **16** | **AGAST** | **36** | **HERR** | **56** | **LZRD** | **76** | **OWLS** |  |  |
| **17** | **BVLVS** | **37** | **ANCH** | **57** | **SNKS** | **77** | **WOODP** |  |  |
| **18** | **MBENTH** | **38** | **NEED** | **58** | **COCO** | **78** | **PASSOMN** |  |  |
| **19** | **SCRUST** | **39** | **KILLI** | **59** | **AMPH** | **79** | **PASSPERD** |  |  |
| **20** | **AMPHI** | **40** | **POEC** | **60** | **L & G** | **80** | **OPSU** |  |  |

**13.** **Mangroves (wet)**


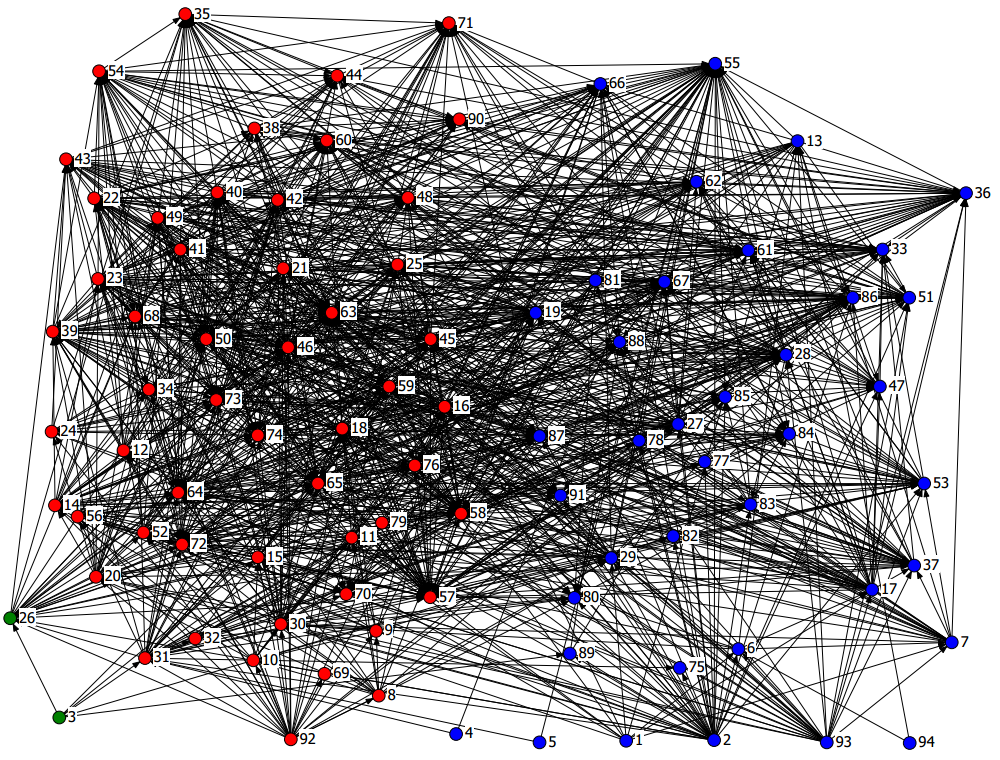


| **ID** | **Name** | **ID** | **Name** | **ID** | **Name** | **ID** | **Name** | **ID** | **Name** |
| --- | --- | --- | --- | --- | --- | --- | --- | --- | --- |
| **1** | **PHY** | **21** | **PENAID** | **41** | **HRSE** | **61** | **PELC** | **81** | **RABT** |
| **2** | **OTH. PP** | **22** | **CARID** | **42** | **SLVR** | **62** | **CORM** | **82** | **SQIUR** |
| **3** | **LEAF** | **23** | **OSHMP** | **43** | **SNOOK** | **63** | **BH & E** | **83** | **M & R** |
| **4** | **WOOD** | **24** | **JLOBST** | **44** | **CUDA** | **64** | **SE & E** | **84** | **FOX** |
| **5** | **ROOT** | **25** | **OCRAB** | **45** | **FWFSH** | **65** | **IBIS** | **85** | **BEAR** |
| **6** | **MICR. H2O** | **26** | **TCRAB** | **46** | **BENTH** | **66** | **DUCK1** | **86** | **RACO** |
| **7** | **ZOOPL.** | **27** | **DCRAB** | **47** | **EFISH** | **67** | **DUCK2** | **87** | **M & O** |
| **8** | **BACT.SED.** | **28** | **PCRAB** | **48** | **SNAP** | **68** | **DUCK3** | **88** | **CATS** |
| **9** | **FLA. SED.** | **29** | **FWINV** | **49** | **MOJA** | **69** | **VULT** | **89** | **DERS** |
| **10** | **CIL. SED.** | **30** | **LARV** | **50** | **SCIAE** | **70** | **K & H** | **90** | **DOLP** |
| **11** | **MEIOF.** | **31** | **INSCT** | **51** | **PIN** | **71** | **MRAPT** | **91** | **MANA** |
| **12** | **MERO** | **32** | **SPIDR** | **52** | **MULL** | **72** | **GUIF** | **92** | **C in SED** |
| **13** | **EPIFN** | **33** | **SHRK** | **53** | **GOBY** | **73** | **SSBIRDS** | **93** | **POC** |
| **14** | **POLY** | **34** | **RAYS** | **54** | **OFISH** | **74** | **G & T** | **94** | **DOC** |
| **15** | **TGAST** | **35** | **TARP** | **55** | **TURT** | **75** | **C & C** |  |  |
| **16** | **AGAST** | **36** | **HERR** | **56** | **LZRD** | **76** | **OWLS** |  |  |
| **17** | **BVLVS** | **37** | **ANCH** | **57** | **SNKS** | **77** | **WOODP** |  |  |
| **18** | **MBENTH** | **38** | **NEED** | **58** | **COCO** | **78** | **PASSOMN** |  |  |
| **19** | **SCRUST** | **39** | **KILLI** | **59** | **AMPH** | **79** | **PASSPERD** |  |  |
| **20** | **AMPHI** | **40** | **POEC** | **60** | **L & G** | **80** | **OPSU** |  |  |

**14.** **Middle Atlantic Bight**


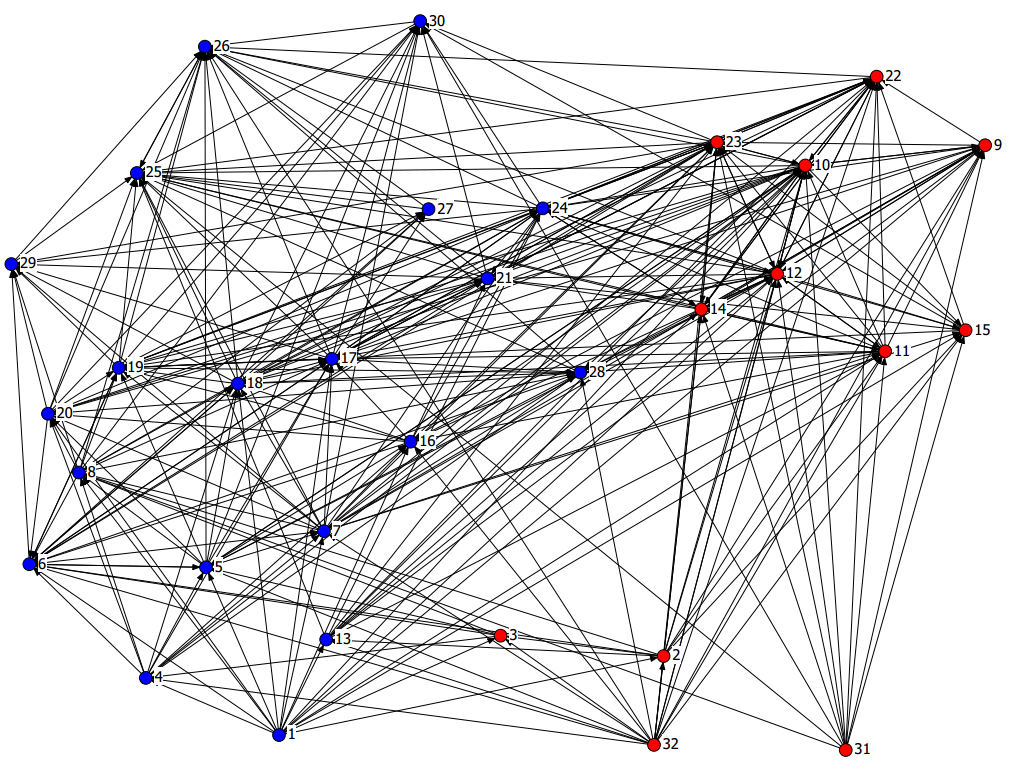


| **ID** | **Name** | **ID** | **Name** | **ID** | **Name** |
| --- | --- | --- | --- | --- | --- |
| **1** | **Phytoplankton- Primary** | **12** | **Macrobenthos- other** | **23** | **Demersals- omnivores** |
| **2** | **Bacteria** | **13** | **Megabenthos- filterers** | **24** | **Demersals- piscivores** |
| **3** | **Microzooplankton** | **14** | **Megabenthos- other** | **25** | **Sharks- coastal** |
| **4** | **Small copepods** | **15** | **Shrimp et al.** | **26** | **Sharks- pelagics** |
| **5** | **Large Copepods** | **16** | **Larval-juv fish- all** | **27** | **Highly Migratory Speci** |
| **6** | **Gelatinous Zooplankton** | **17** | **Small Pelagics- commer** | **28** | **Baleen Whales** |
| **7** | **Micronekton** | **18** | **Small Pelagics- other** | **29** | **Odontocetes** |
| **8** | **Mesopelagics** | **19** | **Small Pelagics- squid** | **30** | **Sea Birds** |
| **9** | **Macrobenthos- polychaet** | **20** | **Small Pelagics- anadro** | **31** | **Discards** |
| **10** | **1Macrobenthos- crustace** | **21** | **Medium Pelagics- (pisc** | **32** | **Detritus- POC** |
| **11** | **Macrobenthos- molluscs** | **22** | **Demersals- benthivores** |  |  |

**15.** **Narragansett Bay**


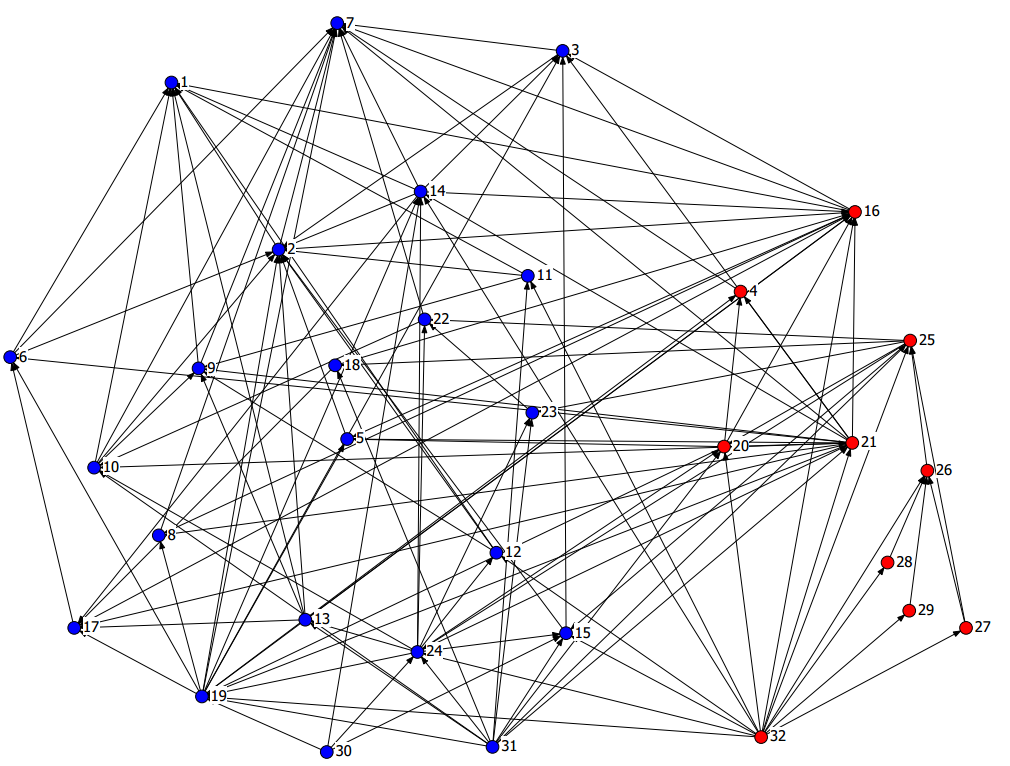


| **ID** | **Name** | **ID** | **Name** | **ID** | **Name** |
| --- | --- | --- | --- | --- | --- |
| **1** | **Bluefish** | **12** | **Bay Anchovy** | **23** | **Fish Larvae** |
| **2** | **Striped Bass** | **13** | **Atl Silversides** | **24** | **Mesozooplankton** |
| **3** | **Winter Flounder** | **14** | **Mummichog** | **25** | **Microzooplankto** |
| **4** | **Windowpane** | **15** | **Hard Clam** | **26** | **Hetero Microflag** |
| **5** | **Scup** | **16** | **Cancer Crabs** | **27** | **Pelag Bacteria** |
| **6** | **Tautog** | **17** | **Am. Lobster** | **28** | **SusPoc Bacteria** |
| **7** | **Dogfish** | **18** | **Softshell Clam** | **29** | **SedPOC Bacteria** |
| **8** | **Skates** | **19** | **Ben Macrofauna** | **30** | **Benthic Alage** |
| **9** | **Longfin Squid** | **20** | **Ben Meiofauna** | **31** | **Phytoplankton** |
| **10** | **Butterfish** | **21** | **Shrimp(Pal+Crg)** | **32** | **Detritus** |
| **11** | **Menhaden** | **22** | **Ctenophores** |  |  |

**16.** **Neuse Estuary (early summer 1997)**


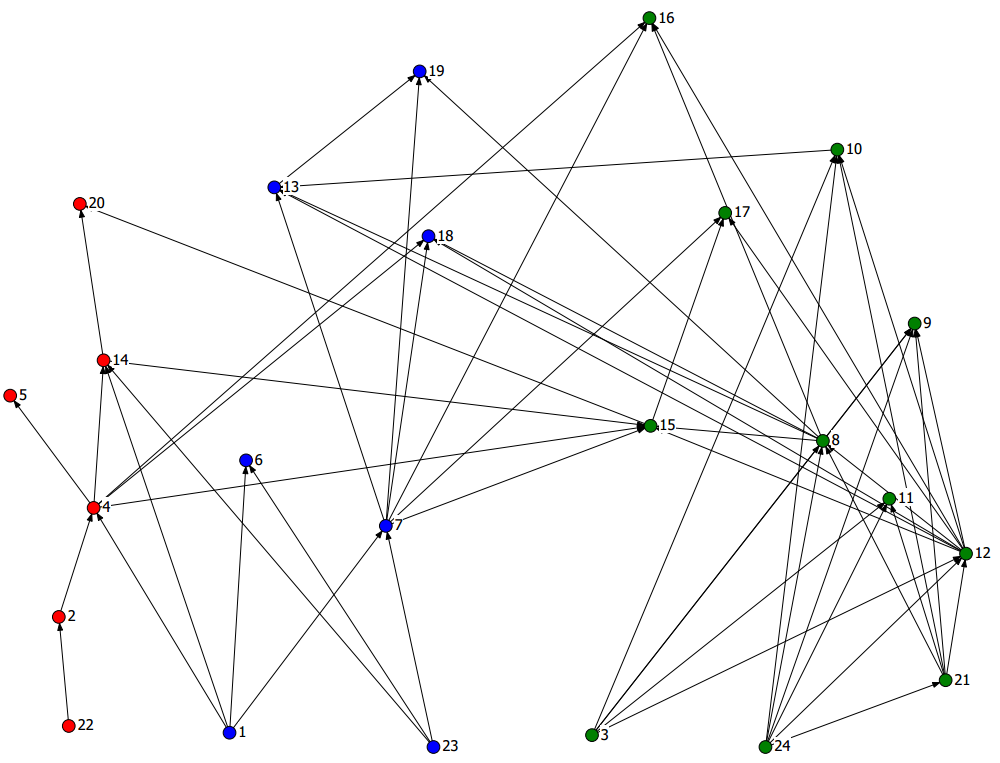


| **ID** | **Name** | **ID** | **Name** | **ID** | **Name** |
| --- | --- | --- | --- | --- | --- |
| **1** | **Phytoplankton** | **9** | **Predatory polychaetes** | **17** | **Blue fish & Flounders** |
| **2** | **Free living bacteria** | **10** | **Deposit feeding amphipods** | **18** | **Birds** |
| **3** | **Benthic microalgae** | **11** | **Isopods** | **19** | **Sea turtles** |
| **4** | **Zooplankton** | **12** | **Meiobenthos** | **20** | **Large predators** |
| **5** | **Jelly fish** | **13** | **Blue crab** | **21** | **Sediment bacteria** |
| **6** | **Oyster** | **14** | **Pelagic fish** | **22** | **DOC** |
| **7** | **Suspension feeding mollus** | **15** | **Pelagic-demersal fish** | **23** | **Suspended POC** |
| **8** | **Deposit feeding polychaet** | **16** | **Demersal fish** | **24** | **Sediment POC** |

**17.** **Neuse Estuary (late summer 1997)**


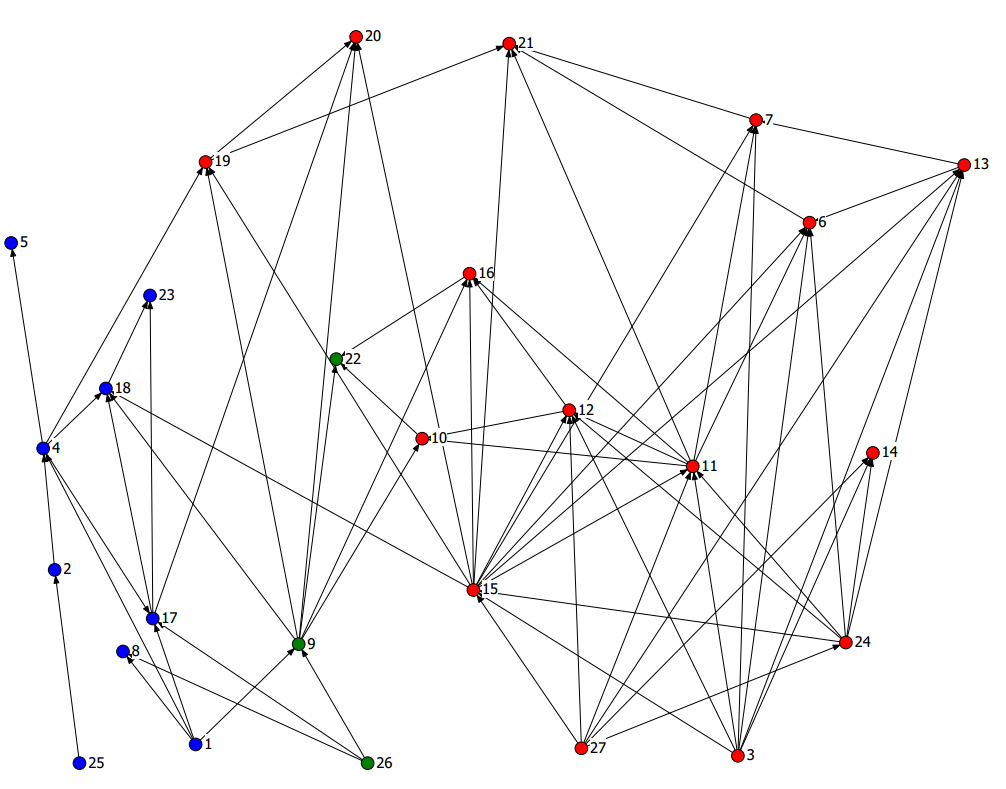


| **ID** | **Name** | **ID** | **Name** | **ID** | **Name** |
| --- | --- | --- | --- | --- | --- |
| **1** | **Phytoplankton** | **10** | **Predatory gastropods** | **19** | **Demersal fish** |
| **2** | **Free living bacteria** | **11** | **Deposit feeding polychaet** | **20** | **Blue fish & Flounder** |
| **3** | **Benthic microalgae** | **12** | **Predatory polychaetes** | **21** | **Birds** |
| **4** | **Zooplankton** | **13** | **Deposit feed. amphipods** | **22** | **Sea turtles** |
| **5** | **Jelly fish** | **14** | **Isopods** | **23** | **Large predators** |
| **6** | **Brown & Pink shrimp** | **15** | **Meiobenthos** | **24** | **Sediment bacteria** |
| **7** | **White shrimp** | **16** | **Blue crab** | **25** | **DOC** |
| **8** | **Oyster** | **17** | **Pelagic fish** | **26** | **Suspended POC** |
| **9** | **Other susp. feed. mollusk** | **18** | **Pelagic-demersal fish** | **27** | **Sediment POC** |

**18.** **Neuse Estuary (early summer 1998)**


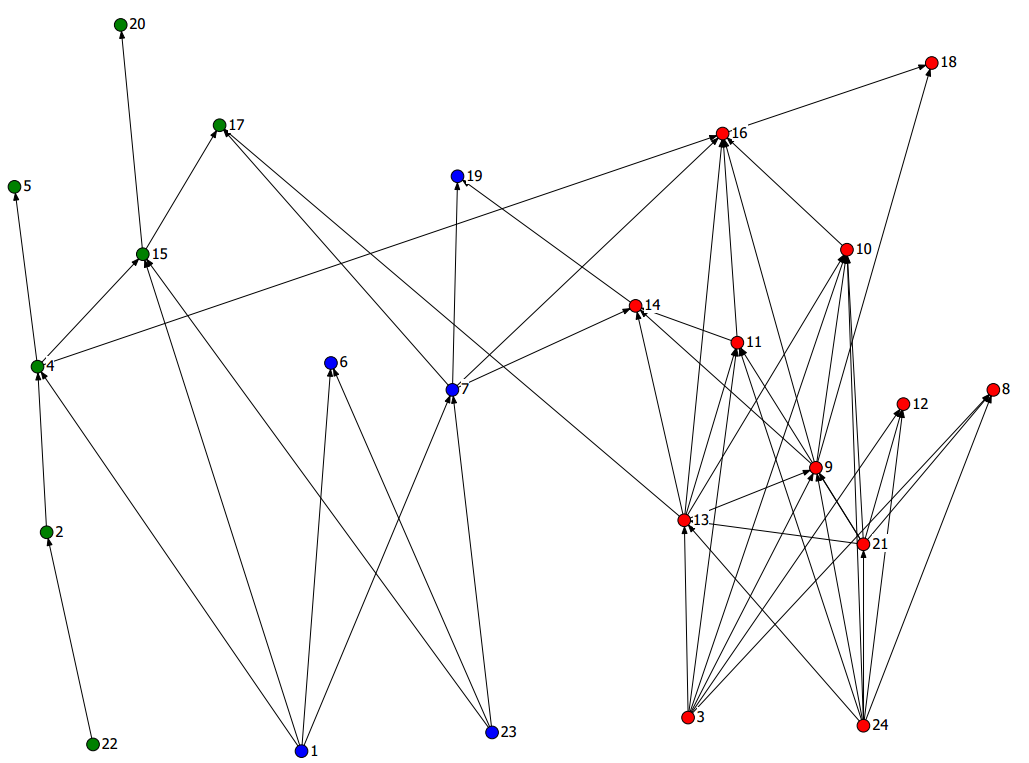


| **ID** | **Name** | **ID** | **Name** | **ID** | **Name** |
| --- | --- | --- | --- | --- | --- |
| **1** | **Phytoplankton** | **9** | **Deposit feed. polychaetes** | **17** | **Blue fish & flounder** |
| **2** | **Free living bacteria** | **10** | **Predatory polychaetes** | **18** | **Birds** |
| **3** | **Benthic microalgae** | **11** | **Deposit feed. amphipods** | **19** | **Sea turtles** |
| **4** | **Zooplankton** | **12** | **Isopods** | **20** | **Large predators** |
| **5** | **Jelly fish** | **13** | **Meiobenthos** | **21** | **Sediment bacteria** |
| **6** | **Oyster** | **14** | **Blue crab** | **22** | **DOC** |
| **7** | **Suspens. feed. mollusks** | **15** | **Pelagic fish** | **23** | **Susp. POC** |
| **8** | **Depost feed. gastropods** | **16** | **Demersal fish** | **24** | **Sed. POC** |

**19.** **Neuse Estuary (late summer 1998)**


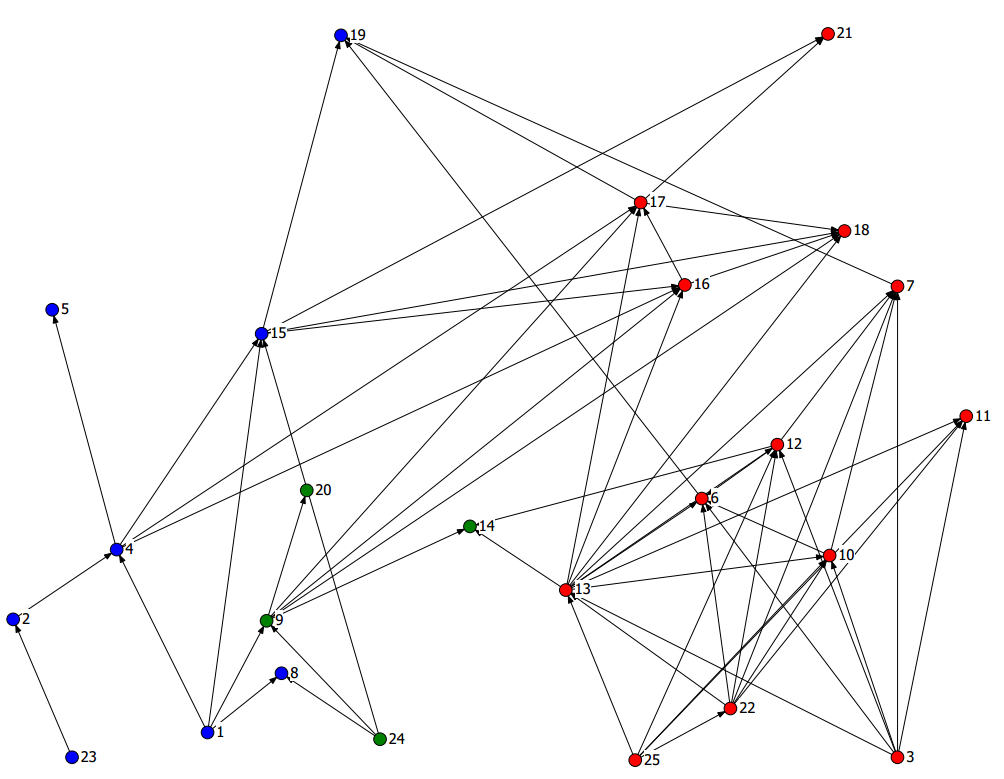


| **ID** | **Name** | **ID** | **Name** | **ID** | **Name** |
| --- | --- | --- | --- | --- | --- |
| **1** | **Phytoplankton** | **10** | **Deposit feed. polychaetes** | **19** | **Birds** |
| **2** | **Free living bacteria** | **11** | **Predatory polychaetes** | **20** | **Sea turtles** |
| **3** | **Benthic algae** | **12** | **Dep. feed. polychaetes** | **21** | **Large predators** |
| **4** | **Zooplankton** | **13** | **Meiobenthos** | **22** | **Sediment bacteria** |
| **5** | **Jelly fish** | **14** | **Blue crab** | **23** | **DOC** |
| **6** | **Brown & Pink shrimp** | **15** | **Pelagic fish** | **24** | **Suspended POC** |
| **7** | **White shrimp** | **16** | **Pelagic-demersal fish** | **25** | **Sediment POC** |
| **8** | **Oyster** | **17** | **Demersal fish** |  |  |
| **9** | **Other susp. feed. mollusk** | **18** | **Blue fish & Flounder** |  |  |

**20.** **Northern Benguela Upwelling**


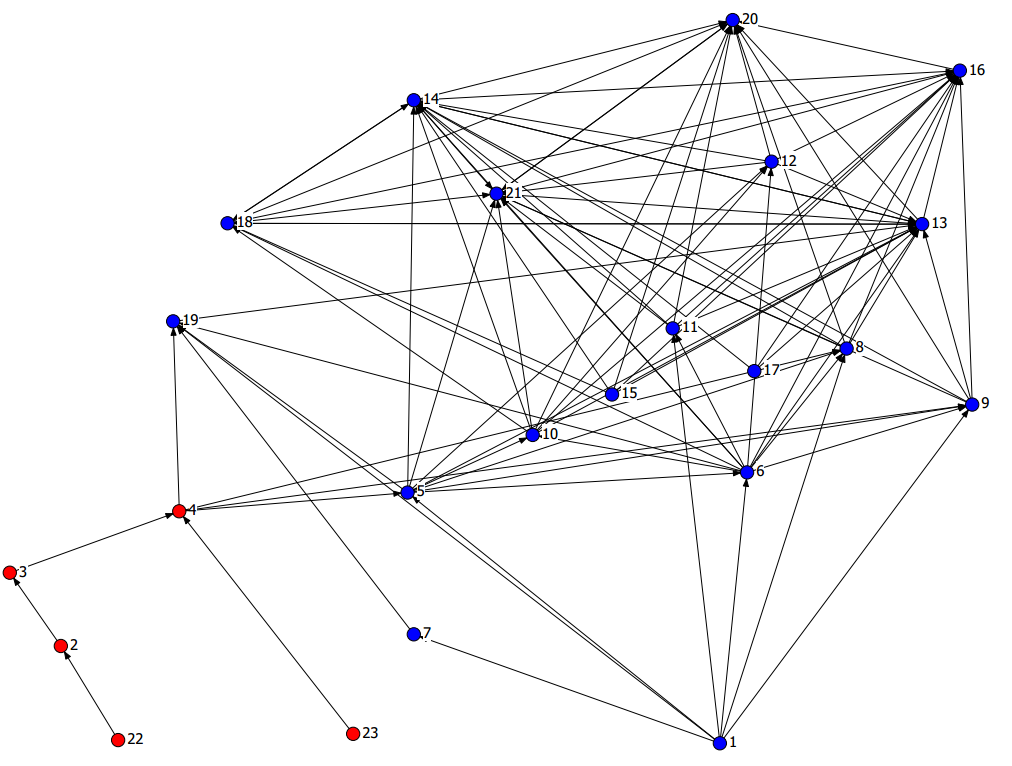


| **ID** | **Name** | **ID** | **Name** | **ID** | **Name** |
| --- | --- | --- | --- | --- | --- |
| **1** | **Phytoplankton** | **9** | **Pilchard** | **17** | **Crab** |
| **2** | **Bacteria** | **10** | **Lanternfish** | **18** | **Squid** |
| **3** | **Flagellates** | **11** | **Gobies** | **19** | **Rock Lobster** |
| **4** | **Microplankton** | **12** | **Horse Mackerel** | **20** | **Seals** |
| **5** | **Mesozooplankton** | **13** | **Hake** | **21** | **Birds** |
| **6** | **Macrozooplankton** | **14** | **Carnivorous Fish** | **22** | **DOC** |
| **7** | **Ichthyofauna** | **15** | **Benthic Fish** | **23** | **POC** |
| **8** | **Anchovy** | **16** | **Snoek** |  |  |

**21.** **Southern New England Bight**


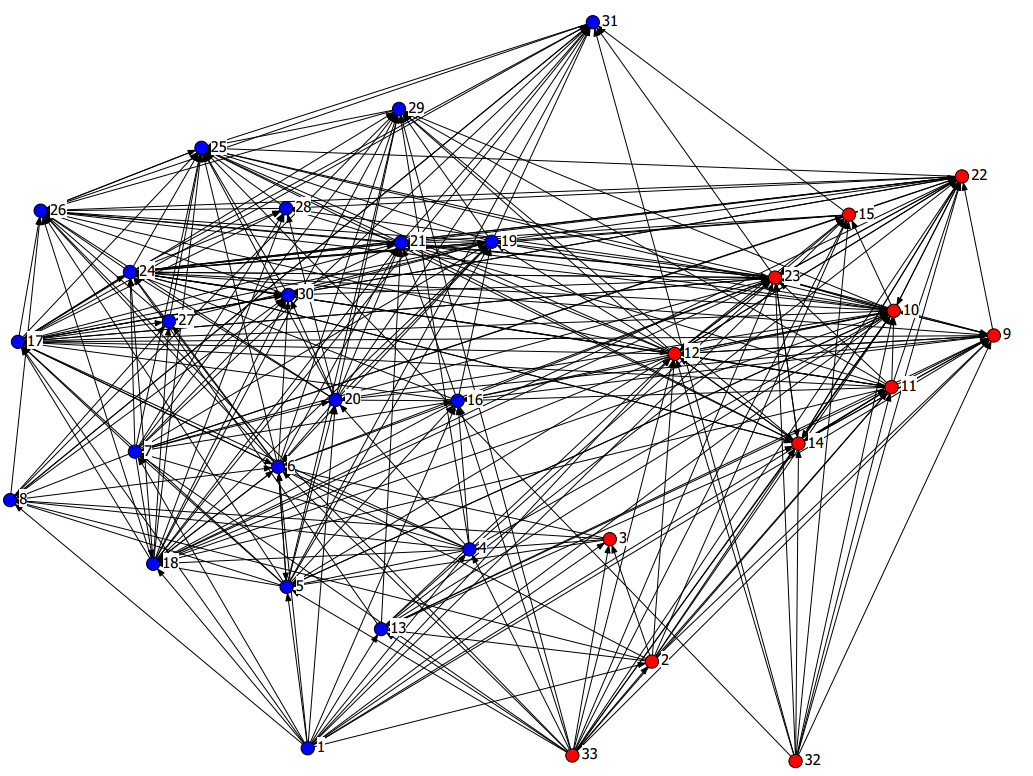


| **ID** | **Name** | **ID** | **Name** | **ID** | **Name** |
| --- | --- | --- | --- | --- | --- |
| **1** | **Phytoplankton- Primary** | **12** | **Macrobenthos- other** | **23** | **Demersals- omnivores** |
| **2** | **Bacteria** | **13** | **Megabenthos- filterers** | **24** | **Demersals- piscivores** |
| **3** | **Microzooplankton** | **14** | **Megabenthos- other** | **25** | **Sharks- coastal** |
| **4** | **Small copepods** | **15** | **Shrimp et al.** | **26** | **Sharks- pelagics** |
| **5** | **Large Copepods** | **16** | **Larval-juv fish- all** | **27** | **Highly Migratory Speci** |
| **6** | **Gelatinous Zooplankton** | **17** | **Small Pelagics- commer** | **28** | **Pinnipeds** |
| **7** | **Micronekton** | **18** | **Small Pelagics- other** | **29** | **Baleen Whales** |
| **8** | **Mesopelagics** | **19** | **Small Pelagics- squid** | **30** | **Odontocetes** |
| **9** | **Macrobenthos- polychaet** | **20** | **Small Pelagics- anadro** | **31** | **Sea Birds** |
| **10** | **Macrobenthos- crustace** | **21** | **Medium Pelagics- (pisc** | **32** | **Discards** |
| **11** | **Macrobenthos- molluscs** | **22** | **Demersals- benthivores** | **33** | **Detritus- POC** |

**22.** **St. Marks Seagrass, site 1 (Jan.)**


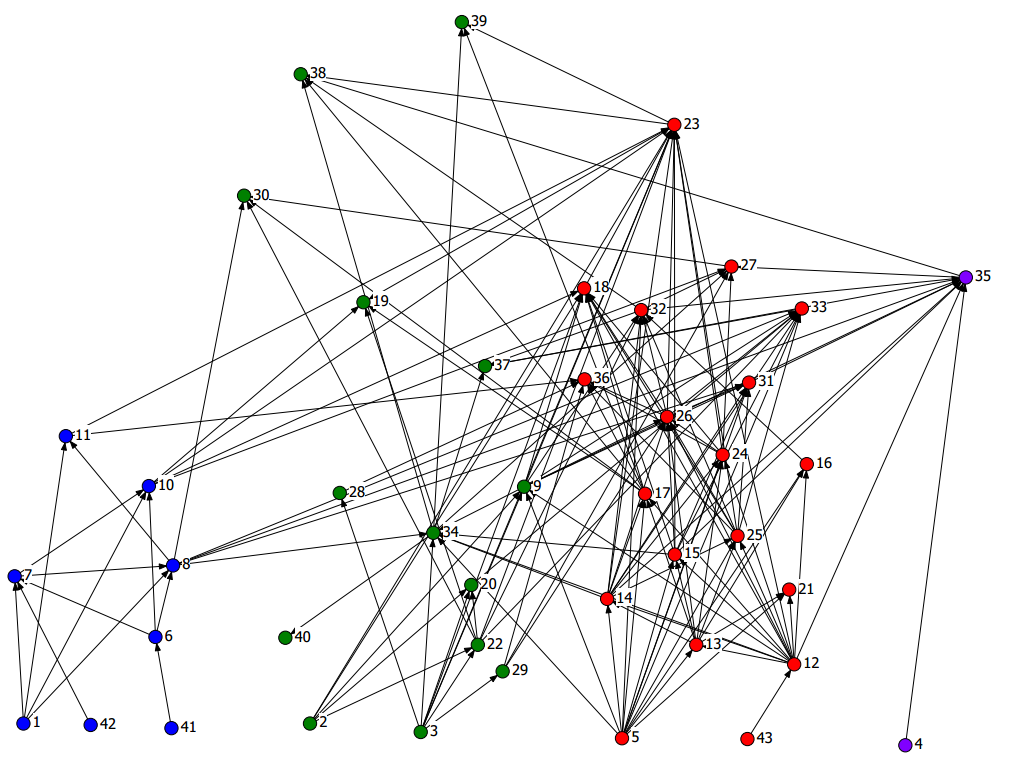


| **ID** | **Name** | **ID** | **Name** | **ID** | **Name** |
| --- | --- | --- | --- | --- | --- |
| **1** | **Phytoplankton** | **16** | **Detritus feed crust.** | **31** | **Pinfish** |
| **2** | **Halodule** | **17** | **Hermit crab** | **32** | **Spot** |
| **3** | **Micro-epiphytes** | **18** | **Omnivorous crabs** | **33** | **Pipefish & seeahorses** |
| **4** | **Macro-epiphytes** | **19** | **Blue crab** | **34** | **Sheepshead minnow** |
| **5** | **Benthic algae** | **20** | **Isopod** | **35** | **Killifish** |
| **6** | **Bacterio plankton** | **21** | **Brittle stars** | **36** | **Benthos-eating birds** |
| **7** | **Micro protozoa** | **22** | **Herbivorous shrimp** | **37** | **Fish-eating birds** |
| **8** | **Zooplankton** | **23** | **Predatory shrimp** | **38** | **Fish & crust. eating bird** |
| **9** | **Epiphyte-graz amphipods** | **24** | **Deposit-feed gastropods** | **39** | **Gulls** |
| **10** | **Suspension-feed molluscs** | **25** | **Deposit-feed polycht** | **40** | **Raptors** |
| **11** | **Suspension-feed polychts** | **26** | **Predatory polycht** | **41** | **DOC** |
| **12** | **Benthic bact** | **27** | **Predatory gastropod** | **42** | **Suspended POC** |
| **13** | **Microfauna** | **28** | **Epiphyte-graz. gastropod** | **43** | **Sediment POC** |
| **14** | **Meiofauna** | **29** | **Other gastropods** |  |  |
| **15** | **Deposit feed amphipods** | **30** | **Southrn hake & sea robins** |  |  |

**23.** **St. Marks Seagrass, site 1 (Feb.)**


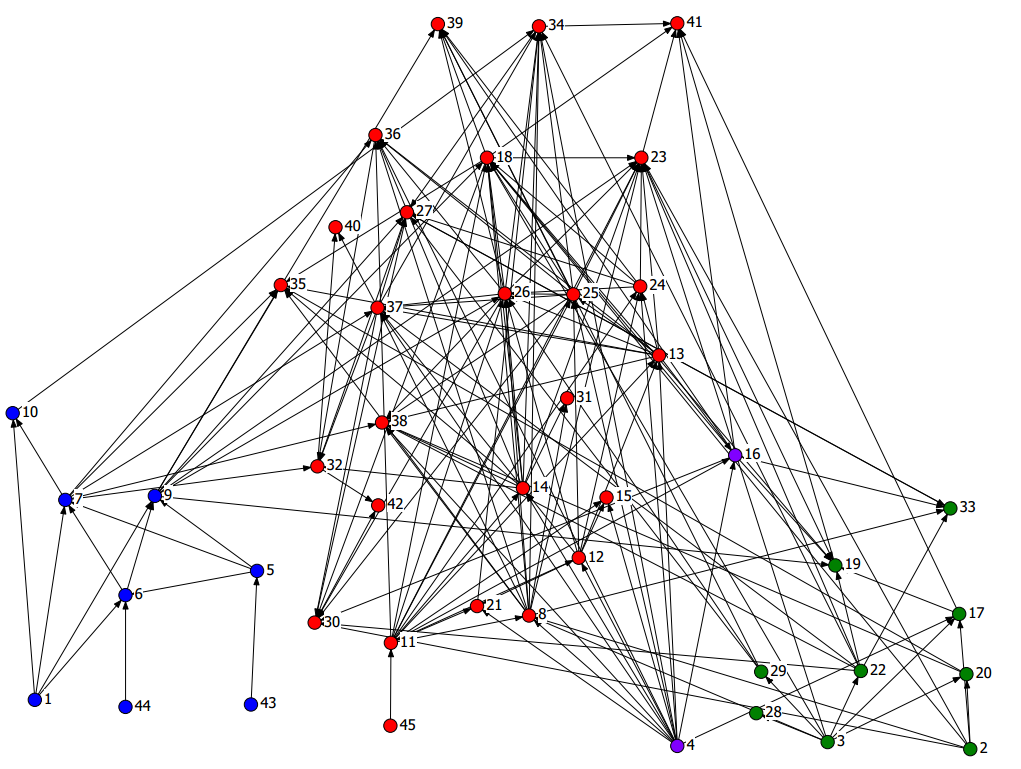


| **ID** | **Name** | **ID** | **Name** | **ID** | **Name** |
| --- | --- | --- | --- | --- | --- |
| **1** | **Phytoplankton** | **16** | **Hermit crab (det)** | **31** | **Tongue fish** |
| **2** | **Halodule** | **17** | **Spider crab (herbiv)** | **32** | **Gulf flound & needle fish** |
| **3** | **Micro-epiphytes** | **18** | **Omnivorous crabs** | **33** | **Southrn hake & sea robins** |
| **4** | **Benthic algae** | **19** | **Blue crab** | **34** | **Atl. silverside & bay anc** |
| **5** | **Bacterio plankton** | **20** | **Isopod** | **35** | **Gobies & blennies** |
| **6** | **Micro protozoa** | **21** | **Brittle stars** | **36** | **Pinfish** |
| **7** | **Zooplankton** | **22** | **Herbivorous shrimp** | **37** | **Spot** |
| **8** | **Epiphyte-graz amphipods** | **23** | **Predatory shrimp** | **38** | **Pipefish & seahorses** |
| **9** | **Suspension-feed molluscs** | **24** | **Deposit-feed gastropod** | **39** | **Benthos-eating birds** |
| **10** | **Suspension-feed polychts** | **25** | **Deposit-feed polycht** | **40** | **Fish-eating birds** |
| **11** | **Benthic bact** | **26** | **Predatory polycht** | **41** | **Gulls** |
| **12** | **Microfauna** | **27** | **Predatory gastropod** | **42** | **Raptors** |
| **13** | **Meiofauna** | **28** | **Epiphyte-graz. gastropod** | **43** | **DOC** |
| **14** | **Deposit feed amphipods** | **29** | **Other gastropods** | **44** | **Suspended POC** |
| **15** | **Detritus feed crust.** | **30** | **Catfish & stingrays** | **45** | **Sediment POC** |

**24.** **St. Marks Seagrass, site 2 (Jan.)**


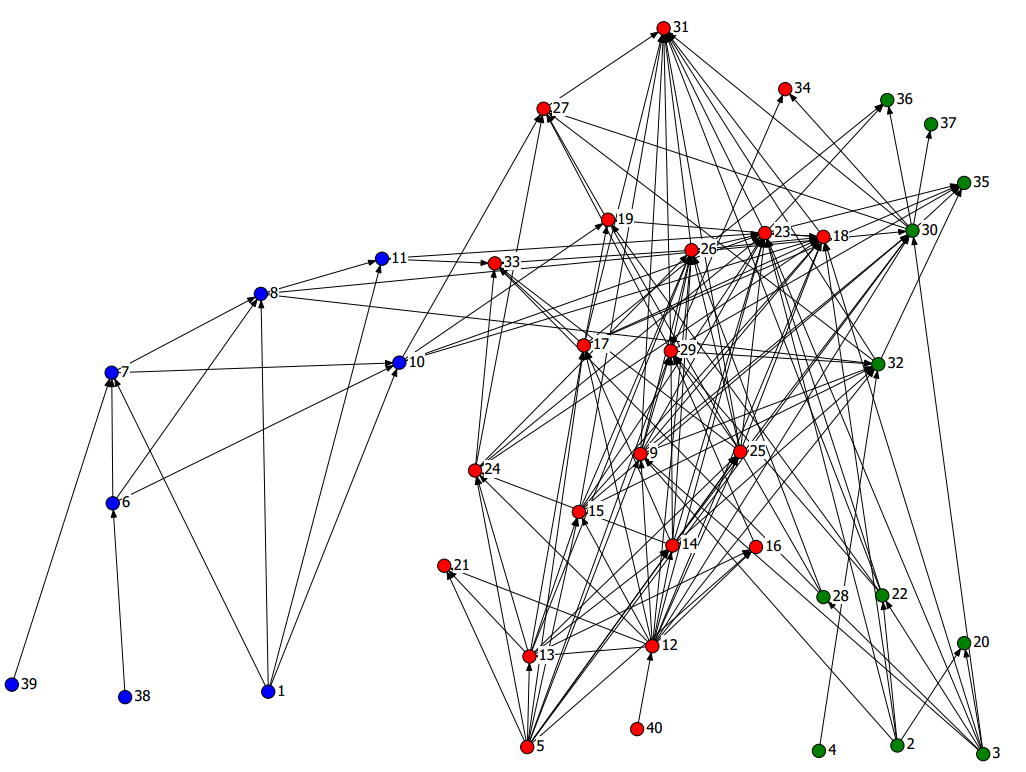


| **ID** | **Name** | **ID** | **Name** | **ID** | **Name** |
| --- | --- | --- | --- | --- | --- |
| **1** | **phytoplankton** | **15** | **deposit feef amphipods** | **29** | **spot** |
| **2** | **Halodule** | **16** | **detritus feed crustacea** | **30** | **sheepshead minnow** |
| **3** | **micro-epiphytes** | **17** | **hermit crab** | **31** | **red drum** |
| **4** | **macro-epiphytes** | **18** | **omniverous crabs** | **32** | **killifish** |
| **5** | **benthic algae** | **19** | **blue crab** | **33** | **benthos eat birds** |
| **6** | **bacterio-plankton** | **20** | **isipods** | **34** | **fish eating birds** |
| **7** | **micro-protozoa** | **21** | **brittle stars** | **35** | **fish & crust eat birds** |
| **8** | **zooplankton** | **22** | **herbivorous shrimp** | **36** | **gulls** |
| **9** | **epiphyte graz amphipods** | **23** | **predatory shrimp** | **37** | **raptors** |
| **10** | **suspension feed molluscs** | **24** | **deposit feed gastropod** | **38** | **DOC** |
| **11** | **suspension feed poplychae** | **25** | **deposit feed polychaete** | **39** | **suspended POC** |
| **12** | **benthic bacteria** | **26** | **predatory polychaete** | **40** | **sediment POC** |
| **13** | **microfauna** | **27** | **predatory gastropod** |  |  |
| **14** | **meiofauna** | **28** | **other gastropods** |  |  |

**25.** **St. Marks Seagrass, site 2 (Feb.)**


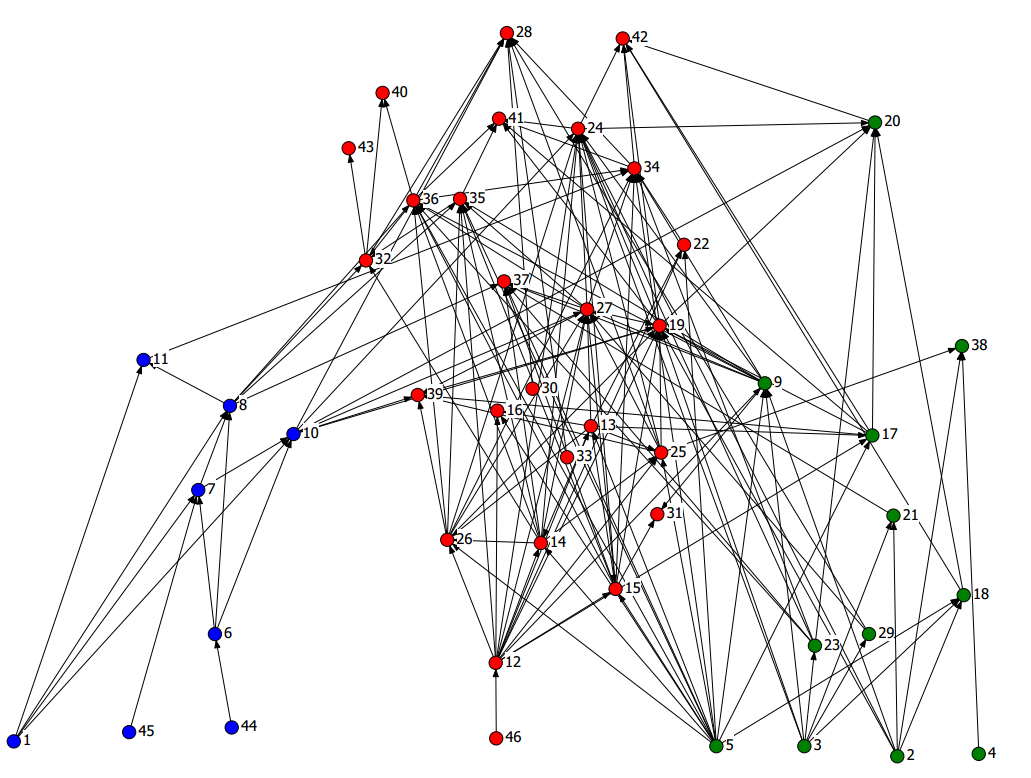


| **ID** | **Name** | **ID** | **Name** | **ID** | **Name** |
| --- | --- | --- | --- | --- | --- |
| **1** | **Phytoplankton** | **17** | **Hermit crab** | **33** | **Southrn hake & sea robins** |
| **2** | **Halodule** | **18** | **Spider crab** | **34** | **Atl. silverside & bay anc** |
| **3** | **Micro-epiphytes** | **19** | **Omnivorous crabs** | **35** | **Pinfish** |
| **4** | **Macro-epiphytes** | **20** | **Blue crab** | **36** | **Spot** |
| **5** | **Benthic algae** | **21** | **Isopod** | **37** | **Pipefish & seahorses** |
| **6** | **Bacterio plankton** | **22** | **Brittle stars** | **38** | **Herbivorous ducks** |
| **7** | **Micro protozoa** | **23** | **Herbivorous shrimp** | **39** | **Benthos-eating birds** |
| **8** | **Zooplankton** | **24** | **Predatory shrimp** | **40** | **Fish-eating birds** |
| **9** | **Epiphyte-graz amphipods** | **25** | **Deposit-feed gastropod** | **41** | **Fish & crust. eating bird** |
| **10** | **Suspension-feed molluscs** | **26** | **Deposit-feed polycht** | **42** | **Gulls** |
| **11** | **Suspension-feed polychts** | **27** | **Predatory polycht** | **43** | **Raptors** |
| **12** | **Benthic bact** | **28** | **Predatory gastropod** | **44** | **DOC** |
| **13** | **Microfauna** | **29** | **Epiphyte-graz. gastropod** | **45** | **Suspended POC** |
| **14** | **Meiofauna** | **30** | **Other gastropods** | **46** | **Sediment POC** |
| **15** | **Deposit feed amphipods** | **31** | **Tongue fish** |  |  |
| **16** | **Detritus feed crust.** | **32** | **Gulf flound & needle fish** |  |  |

**26.** **St. Marks Seagrass, site 3 (Jan.)**


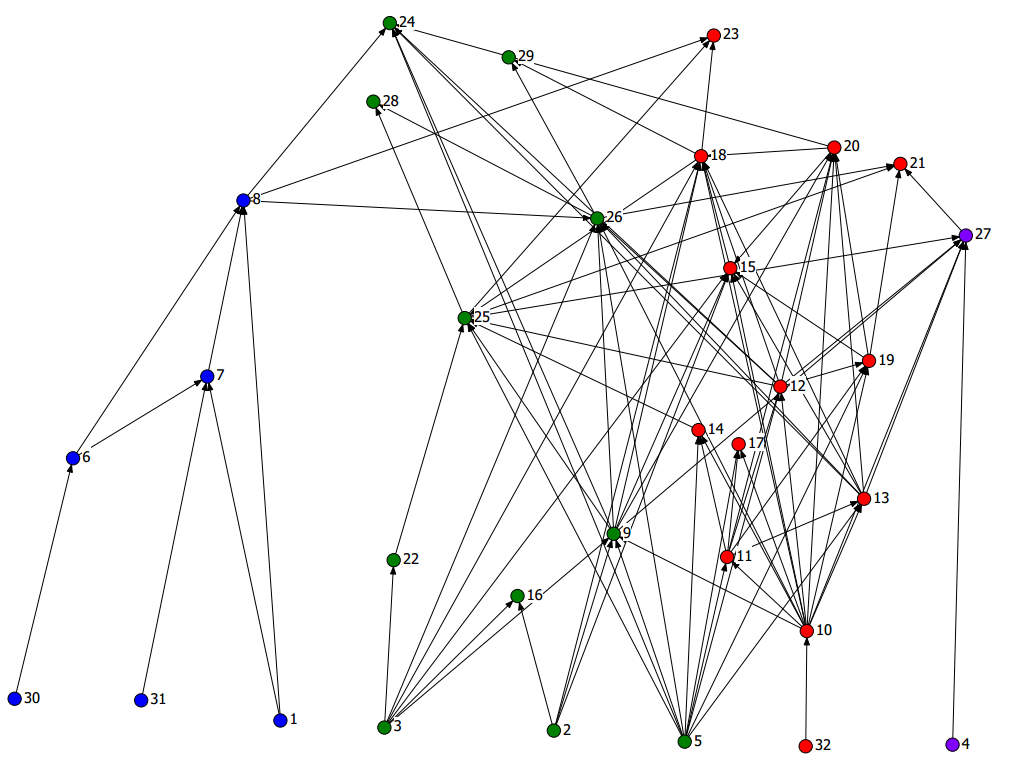


| **ID** | **Name** | **ID** | **Name** | **ID** | **Name** |
| --- | --- | --- | --- | --- | --- |
| **1** | **phytoplankton** | **12** | **meiofauna** | **23** | **southern hake** |
| **2** | **Halodule** | **13** | **deposit feed amphipods** | **24** | **pinfish** |
| **3** | **micro-epiphytes** | **14** | **detritus feed crustaceans** | **25** | **spot** |
| **4** | **macro-epiphytes** | **15** | **omnivorous crabs** | **26** | **sheepshead minnow** |
| **5** | **benthic algae** | **16** | **isopod** | **27** | **killifish** |
| **6** | **bacterio-plankton** | **17** | **brittle stars** | **28** | **fish eating birds** |
| **7** | **micro-protozoa** | **18** | **predatory shrimp** | **29** | **gulls** |
| **8** | **zooplankton** | **19** | **deposit feed gastropod** | **30** | **DOC** |
| **9** | **epiphyte graz amphipods** | **20** | **predatory polychaete** | **31** | **suspended POC** |
| **10** | **benthic bacteria** | **21** | **predatory gastropod** | **32** | **sediment POC** |
| **11** | **microfauna** | **22** | **epiphyte graz gastropod** |  |  |

**27.** **St. Marks Seagrass, site 4 (Feb.)**


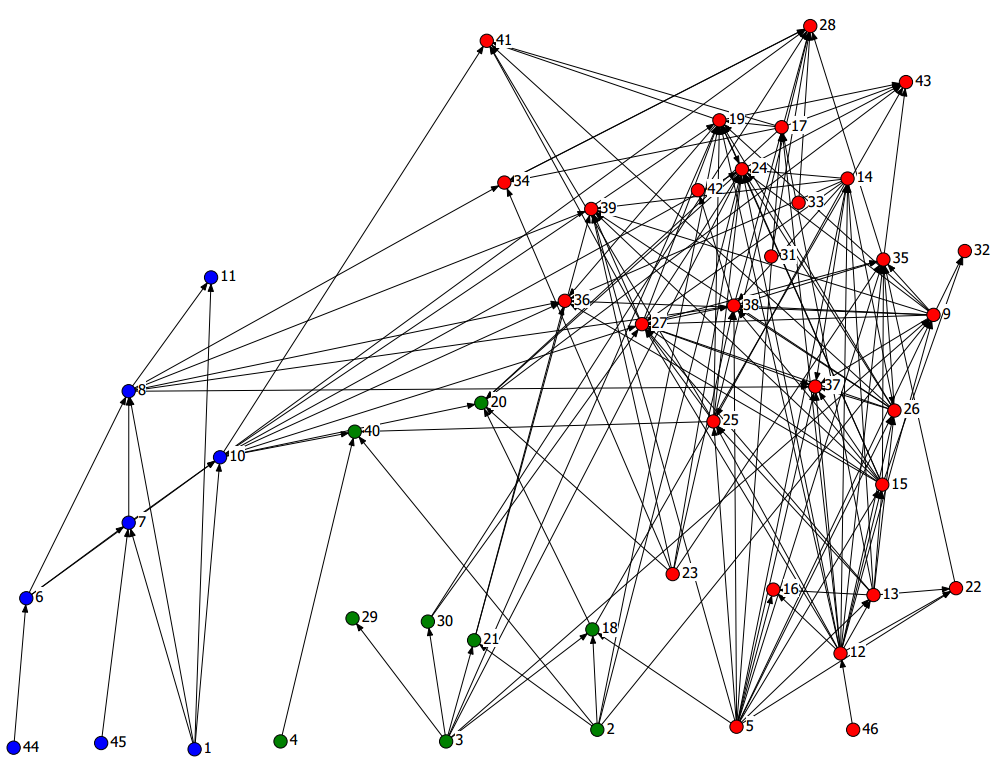


| **ID** | **Name** | **ID** | **Name** | **ID** | **Name** |
| --- | --- | --- | --- | --- | --- |
| **1** | **Phytoplankton** | **17** | **Hermit crab** | **33** | **Gulf flound & needle fish** |
| **2** | **Halodule** | **18** | **Spider crab** | **34** | **Southrn hake & sea robins** |
| **3** | **Micro-epiphytes** | **19** | **Omnivorous crabs** | **35** | **Atl. silverside & bay anc** |
| **4** | **Macro-epiphytes** | **20** | **Blue crab** | **36** | **Gobies & blennies** |
| **5** | **Benthic algae** | **21** | **Isopod** | **37** | **Pinfish** |
| **6** | **Bacterio plankton** | **22** | **Brittle stars** | **38** | **Spot** |
| **7** | **Micro protozoa** | **23** | **Herbivorous shrimp** | **39** | **Pipefish & seeahorses** |
| **8** | **Zooplankton** | **24** | **Predatory shrimp** | **40** | **Herbivorous ducks** |
| **9** | **Epiphyte-graz amphipods** | **25** | **Deposit-feed gastropods** | **41** | **Benthos-eating birds** |
| **10** | **Suspension-feed molluscs** | **26** | **Deposit-feed polycht** | **42** | **Fish-eating birds** |
| **11** | **Suspension-feed polychts** | **27** | **Predatory polycht** | **43** | **Gulls** |
| **12** | **Benthic bact** | **28** | **Predatory gastropod** | **44** | **DOC** |
| **13** | **Microfauna** | **29** | **Epiphyte-graz. gastropod** | **45** | **Suspended POC** |
| **14** | **Meiofauna** | **30** | **Other gastropods** | **46** | **Sediment POC** |
| **15** | **Deposit feed amphipods** | **31** | **Catfish & stingrays** |  |  |
| **16** | **Detritus feed crust.** | **32** | **Tongue fish** |  |  |

**28.** **Sylt-Romo Bight**


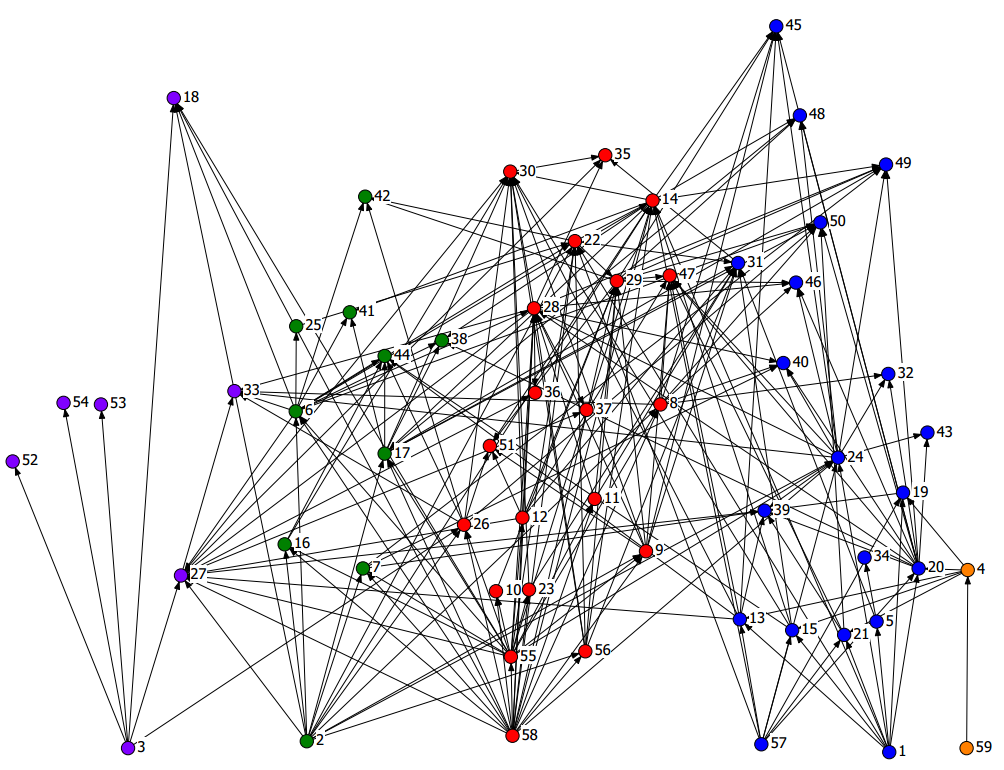


| **ID** | **Name** | **ID** | **Name** | **ID** | **Name** |
| --- | --- | --- | --- | --- | --- |
| **1** | **Phytoplankton** | **21** | **Mya arenaria** | **41** | **Avocet** |
| **2** | **Microphytobenthos** | **22** | **kl polychaetes** | **42** | **Golden Plover** |
| **3** | **Macrophytes** | **23** | **Tharyx killariensis** | **43** | **Knot** |
| **4** | **Freeliving bacteria** | **24** | **Macoma baltica** | **44** | **Dunlin** |
| **5** | **Zooplankton** | **25** | **Phyllodocidae** | **45** | **Bar-tailed Godwit** |
| **6** | **Hydrobia ulva** | **26** | **kl Crustacea** | **46** | **Curlew** |
| **7** | **Littorina littorea** | **27** | **Carcinus maenas** | **47** | **Black-headed Gull** |
| **8** | **Arenicola marina** | **28** | **Crangon** | **48** | **Common Gull** |
| **9** | **Scoloplos amiger** | **29** | **Nepthys** | **49** | **Herring Gull** |
| **10** | **Capitellidae** | **30** | **P. microps [common goby]** | **50** | **Other birds** |
| **11** | **Oligochaeta** | **31** | **P. minutus [common goby]** | **51** | **Mallard** |
| **12** | **Heteromastus** | **32** | **Plaice [P. platessa]** | **52** | **Pintail** |
| **13** | **Lanice conchilega** | **33** | **Flounder [P. flesus]** | **53** | **Widgeon** |
| **14** | **Nereis diversicolor** | **34** | **Herrings** | **54** | **Brent Goose** |
| **15** | **Pygospio elegans** | **35** | **Whiting [M. merlangus]** | **55** | **Sediment bacteria** |
| **16** | **Corophium arenarium** | **36** | **Kabeljauw [G. morhua]** | **56** | **Meiobenthos** |
| **17** | **Corophium volutator** | **37** | **Bull Rout [M. scorpius]** | **57** | **Suspended POC** |
| **18** | **Gammarus spp.** | **38** | **Shelduck** | **58** | **Sediment POC** |
| **19** | **Mytilus edulis** | **39** | **Eider** | **59** | **DOC** |
| **20** | **Cerastoderma** | **40** | **Oystercatcher** |  |  |
